# Cell division and lineage dynamics during antheridium differentiation and male gametophyte development in *Ceratopteris richardii*

**DOI:** 10.1038/s42003-026-10135-w

**Published:** 2026-04-30

**Authors:** Xi Yang, An Yan, Xing Liu, Yun Zhou

**Affiliations:** 1 Department of Botany and Plant Pathology, Purdue University, West Lafayette, IN USA; 2 Purdue Center for Plant Biology, Purdue University, West Lafayette, IN USA; 3 Division of Biology and Biological Engineering, California Institute of Technology, Pasadena, CA USA; 4 Howard Hughes Medical Institute, California Institute of Technology, Pasadena, CA USA; 5 Department of Biochemistry, Purdue University, West Lafayette, IN USA

**Keywords:** Cell fate, Patterning

## Abstract

Sexual reproduction in land plants involves diverse strategies for gamete production and fertilization, significantly differing between seed plants, which deliver sperm via pollen, and seed-free plants such as ferns, which produce motile sperm within specialized multicellular structures, antheridia, on independently growing gametophytes. Despite their crucial roles in sexual reproduction, the cellular mechanisms governing antheridium differentiation and male gametophyte development in ferns remain largely unexplored. Here, using non-invasive, time-lapse confocal imaging combined with computational three-dimensional analysis, we reconstructed detailed lineage maps of antheridium initiation, proliferation, and differentiation in the fern *Ceratopteris richardii*. Our findings demonstrate that antheridium development begins with highly conserved asymmetric cell divisions, giving rise to distinct sterile and spermatogenous cell lineages. Spermatogenous cells undergo synchronized and continuous proliferation, followed by programmed differentiation, eventually forming motile sperm released from mature antheridia. In contrast, the sterile lineage undergoes limited cell divisions, forming structural support tissues surrounding the spermatogenous core. Furthermore, quantitative analyses reveal the cellular basis underlying the previously reported antagonistic effects of the pheromone antheridiogen and abscisic acid on antheridium initiation and spermatogenous cell proliferation. These findings elucidate both conserved and lineage-specific mechanisms regulating sexual differentiation, providing comparative insights into reproductive strategies and hormone-mediated developmental processes across land plants.

## Abstract (web summary)

Time-lapse imaging and three-dimensional analysis reveal how hormone signaling coordinates asymmetric cell division and lineage specification during antheridium formation and male gametophyte development in the fern *Ceratopteris richardii*.

## Introduction

Sexual reproduction is essential for offspring production and genetic diversity in land plants^1^. In seed plants, the life cycle is dominated by the sporophyte generation, which is initiated through fertilization of egg cells by sperm^2,3^. The gametophytes of seed plants are highly reduced, physically embedded within, nutritionally dependent on the sporophyte^1,4,5^. Fertilization occurs through sperm delivered by pollen (male gametophytes) to egg cells located within embryo sacs (female gametophytes)^6–9^. In contrast, ferns, vascular plants that reproduce via spores instead of seeds, possess free-living gametophytes that grow independently of their sporophytes^10,11^. Fern gametophytes differentiate specialized reproductive organs: sperm-producing antheridia and egg-bearing archegonia, which are crucial for gamete formation and successful fertilization^11^. Unlike pollen-based sperm production and delivery in seed plants, fern sperm form and mature within antheridia; upon maturation, motile sperm are released from the antheridia and swim to archegonia for fertilization^4,11,12^. The unique and specialized multicellular structures of antheridia in fern gametophytes have fascinated botanists and developmental biologists for more than 150 years. Early foundational observations and illustrations of antheridium formation in ferns were made by pioneers such as L. Kyn^13^ and D. Campbell^14^. A significant breakthrough occurred in 1950, when Döpp discovered that immature gametophytes of the fern *Pteridium aquilinum* developed antheridia when cultured in media previously conditioned by other gametophytes. This finding suggested that a diffusible phytohormone, later named antheridiogen, triggers antheridium development^15^. Subsequent studies by multiple research groups worldwide demonstrated that antheridiogen is a natural pheromone produced and secreted by female or hermaphroditic gametophytes, capable of inducing antheridium formation, sometimes with cross-species activity^16–22^. Antheridiogens isolated in fern species from several different families have since been identified as gibberellic acids (GAs) or GA-like molecules^23–25^. These discoveries positioned antheridia as a model for studying hormone-mediated developmental transitions.

Over the past a few decades, the fern *Ceratopteris richardii* has been established as a model organism for studying not only fern development but also broader questions related to gene function and the evolution of vascular plants^11,26–37^. During the haploid gametophyte phase, Ceratopteris exhibits two distinct sex types: hermaphrodites and males^11,27^. Hermaphrodites develop only a few antheridia, typically distant from the meristem and egg-producing archegonia. In contrast, males, under the influence of antheridiogen, continuously produce antheridia at various developmental stages throughout the prothallus^27,38^, providing a powerful system to investigate the regulation of antheridium initiation and differentiation. Previous anatomical and microscopic studies, including transverse sections, histological staining, scanning electron microscopy (SEM), and transmission electron microscopy (TEM) of fixed antheridia at various developmental stages, have revealed that each developing antheridium in Ceratopteris males consists of spermatogenous cells and surrounding sterile tissues^12,38,39^. In Ceratopteris, the spermatogenous cells are located within the center of the antheridium, where they undergo division and differentiation to eventually produce 32 sperm cells (male gametes)^39^; the sterile tissue forms cap and ring cells located externally^12,38^, enclosing the spermatogenous core and providing structural support and protection. The organization of the spermatogenous core and surrounding sterile tissue appears to be conserved across many fern species, although the basal sterile layer of antheridia has been variably described in the literature as either a basal shield cell or an additional ring cell, depending on the species and study^39–41^. In addition to the role of antheridiogen, Hickok reported that the phytohormone abscisic acid (ABA) antagonized the effects of antheridiogen in Ceratopteris, blocking antheridium formation^42^. Subsequent genetic screens identified ABA-insensitive mutants in Ceratopteris^43^. Importantly, McAdam et al. (2016) described a mutant carrying a lesion in the *Ceratopteris* homolog of *OST1*, a conserved component of the SnRK2 signaling pathway, providing genetic evidence that ABA signaling contributes to sex determination in *Ceratopteris*^44^.

Despite this understanding, the dynamics of cell division and lineage alterations during antheridium development and spermatogenesis in Ceratopteris gametophytes remain poorly understood. Additionally, the cellular mechanisms underlying antheridium initiation and proliferation in response to environmental cues are not known. Here, we performed non-invasive, time-lapse confocal imaging to capture the dynamic processes of antheridium initiation and maturation. Using computational image analysis, we reconstructed detailed lineage maps of antheridium formation and male gametophyte development in four dimensions (3D plus time), uncovering distinct developmental trajectories and cell division activities within sterile and spermatogenous lineages. Quantitative imaging and mutant analyses further revealed immediate effects of antagonistic phytohormones on antheridium cell division and differentiation. These findings clarify cell behaviors and underlying principles of male gametophyte development in seed-free vascular plants, providing insights into the evolution and regulatory mechanisms of gametogenesis and spermatogenesis in land plants.

## Results

### Development of antheridia and male gametophytes in Ceratopteris

**Fig. 1:**
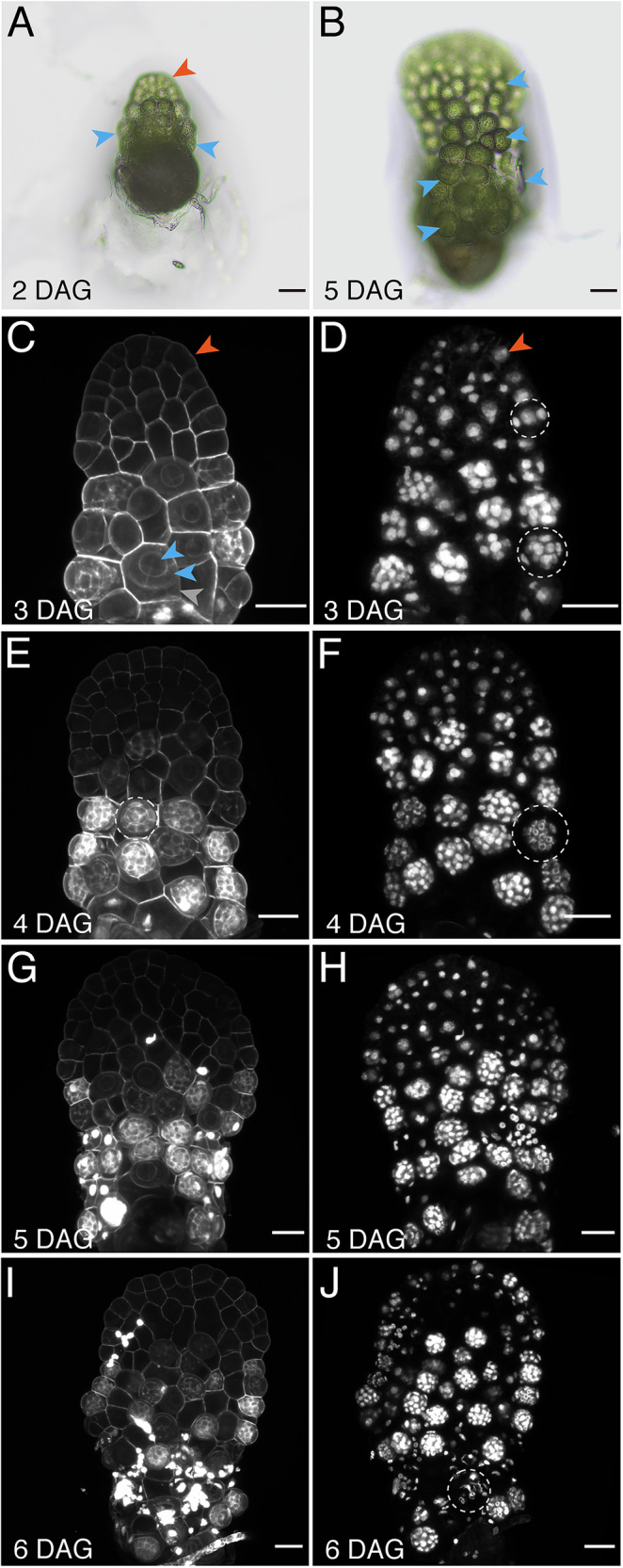
Light and confocal imaging of Ceratopteris male gametophytes at different developmental stages. **A**, **B** Light micrographs of a Ceratopteris male gametophyte grown in the presence of antheridiogen at two days after germination (2 DAG) (**A**) and 5 DAG (**B**). **C**–**J** Z-projection views of confocal images of different wild-type male gametophytes taken at 3 DAG (**C**, **D**), 4 DAG (**E**, **F**), 5 DAG (**G**, **H**), and 6 DAG (**I**, **J**). **C**, **E**, **G**, **I** Confocal images showing cell outlines of four male gametophytes. **D**, **F**, **H**, **J** Confocal images of the same gametophytes shown in (**C**, **E**, **G**, **I**), with the stain highlighting nuclei (See Methods for details). Blue arrowheads in (**A**, **B**) and dashed circles in (**D**, **E**, **F**, **J**) indicate antheridia at different developmental stages; orange arrowheads (**A**, **C**, **D**) indicate non-antheridium cells; blue arrowheads (**C**) indicate sterile cells including cap and ring cells; and gray arrowheads (**C**) indicate basal shield cells. Scale bars: 50 μm. At least three samples were examined at each indicated stage, showing comparable results.

We first characterized the developmental progression of Ceratopteris male gametophytes (Fig. 1A-B). Wild-type (WT) Hn-n spores were surface-sterilized and spread on conditioned fern media (CFM), which contains antheridiogen to promote male differentiation (see Methods for details). At two days after germination (2 DAG), cells in the basal half of the male gametophyte began differentiating into antheridia (blue arrowheads), while cells in the apical half remain undifferentiated (orange arrowhead, Fig. 1A). By 5 DAG, the prothallus of the same male gametophyte had expanded, and cells in the apical half also began initiating antheridium differentiate. At this stage, nearly all cells in the basal half had differentiated into antheridia, and several early-formed antheridia had matured and ruptured (blue arrowheads, Fig. 1B). To better examine the cellular organization of developing antheridia, we stained male gametophytes at different DAGs and imaged them using laser scanning confocal microscopy (Fig. 1C-J). Each sample was first stained to visualize cell outlines (Fig. 1C, E, G, I), followed by nuclear staining of the same specimen (Fig. 1D, F, H, J). These analyses revealed the multicellular structure of antheridia, which was distinct from non-antheridium cells. Non-antheridium cells exhibited a typical rectangular or trapezoid shape (Fig. 1C, E, G, I; one example highlighted by an orange arrowhead in Fig. 1C) and contained a single nucleus per cell (Fig. 1D, F, H, J; orange arrowhead in Fig. 1D). In contrast, cell wall staining showed that early-stage antheridia formed a three-dimensional dome-like structure composed of three layers (blue and gray arrowheads, Fig. 1C), consistent with previous observations^12,38^. From top to bottom, the upper two layers are referred to as the cap and ring cells (blue arrowheads in Fig. 1C, as labelled in ref. ^38^). The third, bottom layer, referred to here as the basal shield cell (gray arrowhead, Fig. 1C), was previously described as an additional ring cell in another study^12^. We use the term “basal shield cell” to emphasize its position at the base of the antheridium, its connection to the gametophyte body, and to distinguish it from the ring cell in the middle layer (blue arrowhead, Fig. 1C). As development progressed, a number of small, adjacent spermatogenous cells appeared at the center of each antheridium (dashed circle, Fig. 1D), surrounded by sterile cap and ring cells. Nuclear staining provided further insight into the internal organization of antheridia. Early-stage antheridia contained only two to a few nuclei, whereas late-stage antheridia exhibited multiple densely packed nuclei at the center (dashed circles, Fig. 1D). These nuclei correspond to developing sperm cells, as each antheridium ultimately produces 32 sperm^39^. In mature antheridia, many of these nuclei underwent nuclear coiling (dashed circle, Fig. 1F), likely reflecting the second phase of spermatogenesis, indicating the proliferation of spermatogenous cells followed by spermiogenesis. Additionally, ruptured antheridia and motile spermatozoids with tightly coiled nuclei were captured by staining and confocal imaging (dashed circle, Fig. 1J). Interestingly, the distinct morphologies of antheridia within each male gametophyte allowed us to define their differentiation stages. These observations suggested that antheridium initiation typically began along the lateral margins of the basal half of the prothallus. Subsequently, additional antheridia formed more randomly throughout the basal region and gradually appeared along the lateral margins of the apical half, while the apical tip remained undifferentiated or was the last to differentiate (Fig. 1C-J).

**Fig. 2:**
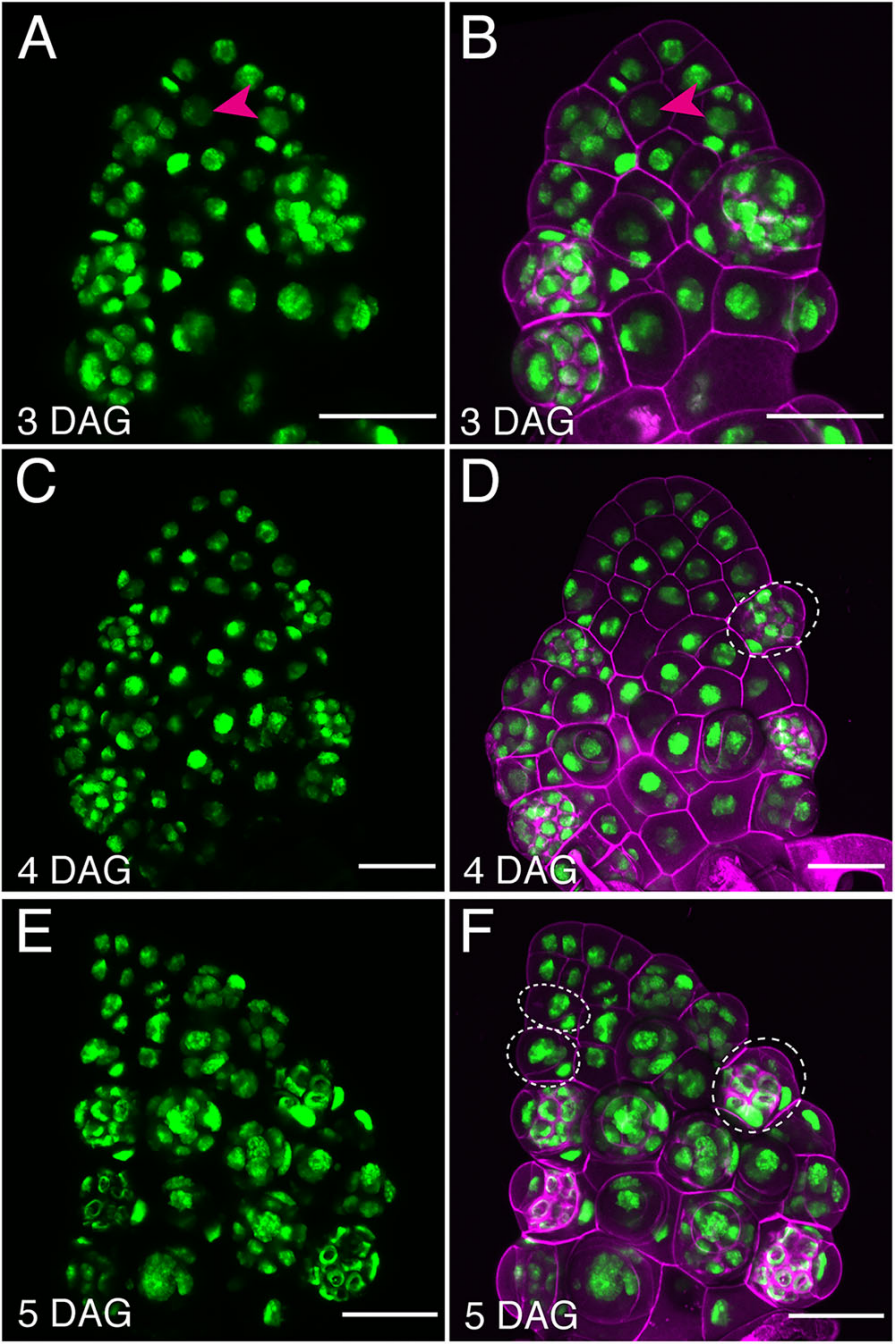
Confocal imaging of nuclei and cellular structures in Ceratopteris male gametophytes at different developmental stages. **A**–**F** Z-projection views of male gametophytes expressing the *pCrUBQ10::H2B-GFP::3’CrUBQ10* nuclear marker from 3 to 5 DAG. **A**, **C**, **E** GFP channel showing nuclear localization. **B**, **D**, **F** Merged channels of GFP (green) and PI counterstain (showing cell outlines, magenta). Magenta arrowheads indicate an antheridium mother cell. White dashed circles highlight antheridia at different developmental stages, with zoomed-in views shown in Fig. S1. Scale bars: 50 μm. At least three samples were examined at each indicated stage, showing comparable results.

**Fig. 3:**
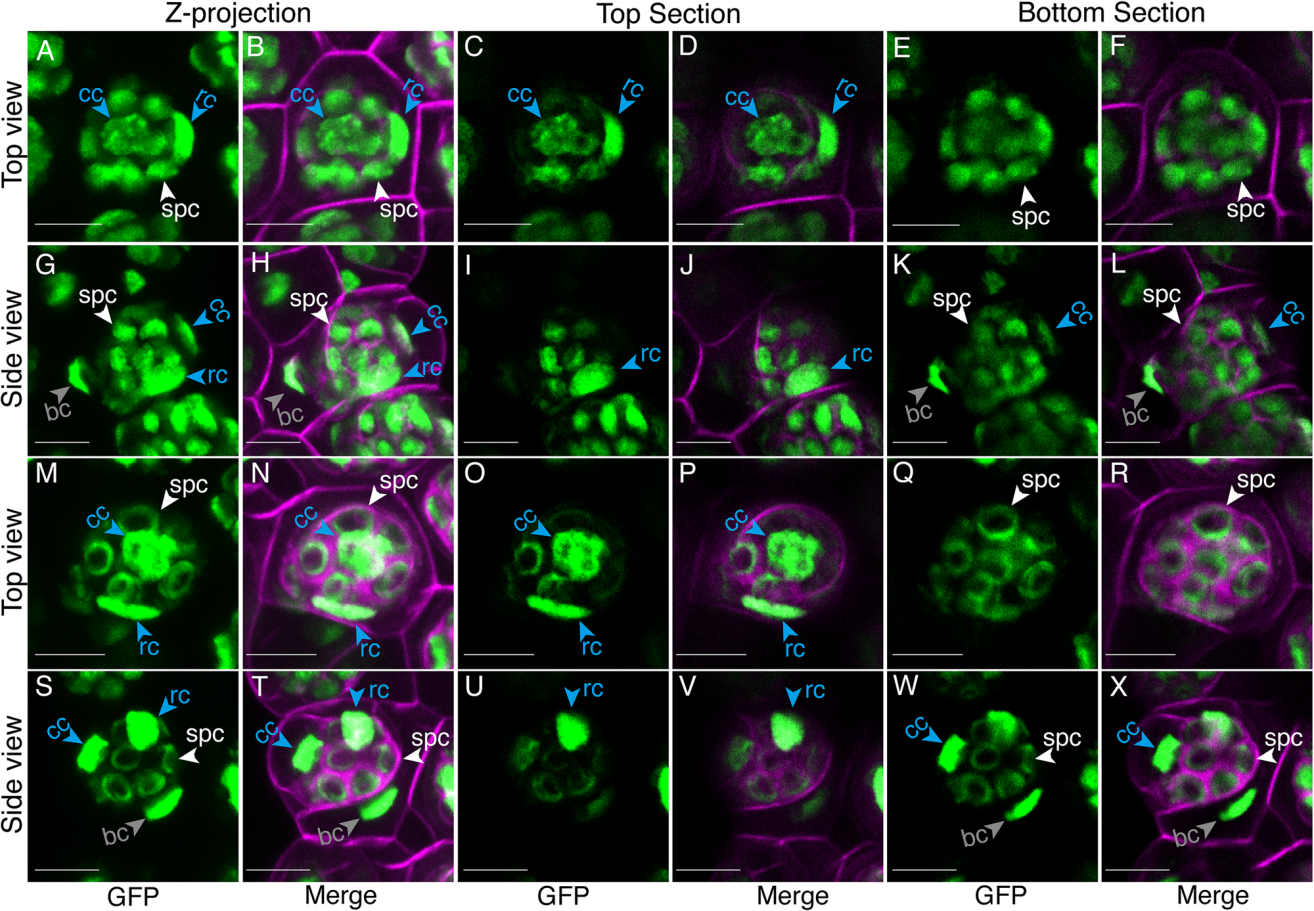
Confocal imaging of nuclear and cellular organization in Ceratopteris antheridia before and during sperm differentiation. **A**–**L** Two representative antheridia imaged prior to sperm differentiation, shown from a top view (**A**–**F**) and a side view **G**–**L**, with spermatogenous cell nuclei appearing compact and solid. **M**–**X** Two representative antheridia undergoing sperm differentiation, shown from a top view (**M**–**R**) and a side view (**S**–**X**), with nuclei forming characteristic ring-like structures. **A**, **B**, **G**, **H**, **M**, **N**, **S**, **T** Z-projection views of individual antheridia. **C**–**F**, **I**–**L**, **O**–**R**, **U**–**X**) Transverse sectional views taken from both upper (top) and lower (bottom) positions, revealing nuclear organization within the 3D structure of the antheridia. Blue arrowheads indicate cap cells (cc) and ring cells (rc); gray arrowheads indicate basal shield cells (bc); and white arrowheads indicate spermatogenous cells (spc; **A**–**L**) or differentiating sperm cells (spc; **M**–**X**). Scale bars: 20 μm. At least three samples were examined at each indicated stage, showing comparable results.

To further examine the stages of antheridium cell division and differentiation at high resolution, we imaged male gametophytes from the Ceratopteris transgenic line ubiquitously expressing a nuclear marker (*pCrUBQ10::H2B-GFP::3’CrUBQ10*)^45^. Confocal imaging results showed both nuclei (green) and cell walls (magenta) in gametophytes at different DAGs (Figs. 2A-F, S1A-D), aligning with our earlier observations using the sequential staining method (Fig. 1C-J). More importantly, these images enabled us to identify distinct stages of antheridium development, beginning with the one-nucleate antheridium mother cell (magenta arrowheads, Fig. 2A, B). A subsequent stage showed two nuclei: one antheridium initial cell (yellow arrowhead, Fig. S1A) and one basal shield cell (gray arrowhead, Fig. S1A). We also observed an early-stage antheridium containing three nuclei corresponding to a sterile cell, a spermatogenous cell, and a basal shield cell (blue, white, and gray arrowheads, respectively, Fig. S1B). At later developmental stages, antheridia exhibited two sterile cells (blue arrowheads, corresponding to the previously defined cap and ring cells^38^), multiple spermatogenous cells (white arrowheads), and the basal shield cell (gray arrowhead) (Fig. 2D and S1C). We also captured antheridia undergoing sperm differentiation (Figs. 2F and S1D), characterized by sterile cap and ring cells (blue arrowheads), spermatozoids with coiled nuclei (white arrowheads), and a basal shield cell (gray arrowheads). Based on these observations, we compared antheridia at the two late distinct developmental stages (Fig. 3A-X): antheridia that had completed or nearly completed spermatogenous cell proliferation but had not yet initiated sperm differentiation, and antheridia had completed spermatogenous cell division and were undergoing sperm differentiation, prior to antheridium rupture and sperm release. Antheridia from both stages were stained and imaged from top and side orientations, and z-projection and optical transverse sectional views were generated from confocal stacks along the z-axis to resolve antheridium morphology as well as nuclear and cellular organization (Fig. 3A-X). Before sperm differentiation (Fig. 3A-L), spermatogenous cells were arranged in multiple well-organized layers at the center of the antheridium, with GFP-labeled nuclei appearing compact and solid (white arrowheads). In contrast, following completion of spermatogenous cell division and initiation of sperm differentiation (Fig. 3M-X), developing sperm cells exhibited characteristic coiled, ring-like nuclei while remaining centrally positioned within layered cell arrangement (white arrowheads). Taken together, these high-resolution snapshots of antheridia and male gametophytes across developmental stages (Figs. 2–3, S1) reveal the progression from antheridium initiation through cell proliferation and differentiation in Ceratopteris.

### Time-lapse imaging reveals key steps and cell division dynamics during antheridium formation and male gametogenesis

**Fig. 4:**
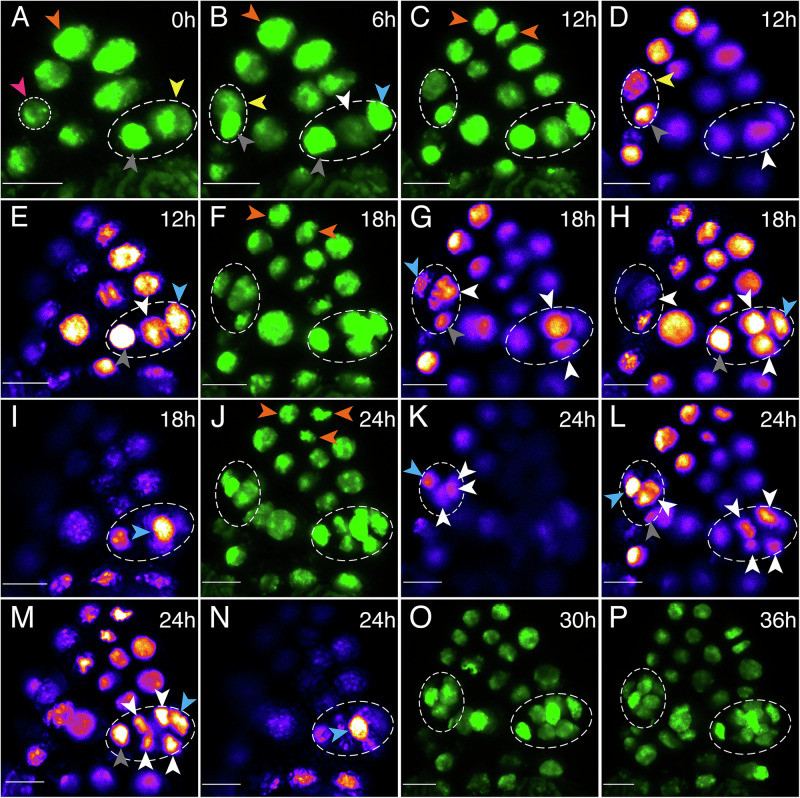
Time-lapse confocal imaging reveals cell dynamics during male gametophyte development in response to antheridiogen. **A**–**P** Confocal images of a Ceratopteris male gametophyte expressing the *pCrUBQ10::H2B-GFP::3’CrUBQ10* transgenic reporter. **A** At 1 DAG, a male gametophyte grown on conditioned fern medium (CFM, containing antheridiogen) was live-imaged by laser scanning confocal microscopy as the initial time point (0 h). The gametophyte was grown on CFM and subsequently imaged every six hours. Images from 0 h (**A**) to 36 h (**P**) are shown in this figure, and images from later time points (42–72 h) are present in Fig. S2. White dashed circles highlight the developmental progression of the first- and second-formed sperm-producing antheridia. Magenta arrowheads indicate antheridium mother cells; orange arrowheads indicate a non-antheridium cell and its progeny at the apical region of the gametophyte; yellow arrowheads indicate antheridium initial cells; gray arrowheads indicate basal cells; blue arrowheads indicate sterile cells including cap cells (**H**, **K**, **M**) and ring cells (**I**, **L**, **N**); and white arrowheads indicate spermatogenous cells. **A**–**C**, **F**, **J**, **O**, **P** Z-projection views of GFP signals (green) from 0 to 36 h. **D**, **E**, **G**–**I**, **K**–**N** Transverse sectional views of GFP signals (Fire LUT) at different positions along the Z-axis from 12-24 h, visualizing nuclei within the 3D structures of developing antheridia. Scale bars (**A**–**P**): 20 μm. At least three samples were live-imaged under the same conditions and time frames, showing comparable results. One representative sample (Sample 9) is shown here, with two independent replicates included in Fig. S3 and Fig. S4.

Next, to define sequential steps of antheridium initiation and proliferation and to examine the cellular dynamics underlying male gametophyte development, we performed non-invasive confocal time-lapse imaging using the same nuclear marker (Figs. 4A-P, S2-S4). Reporter spores were spread on CFM to induce specification and development of male gametophytes after spore germination. At one day after germination (DAG), male gametophytes were transferred to fresh CFM to ensure continuous exposure to antheridiogen and imaged directly on CFM plates using confocal microscopy without mounting or staining to establish the initial time point (0 h, 0 h) (Figs. 4A, S3A, and S4A). After each imaging session, gametophytes were returned to identical growth conditions, and live imaging was performed every six hours until the early-formed antheridia had fully developed, containing a large number of spermatozoids and ready for release into the environment (Figs. 4B-P, S2A-G, S3B-N, S4B-N).

Time-lapse imaging revealed two distinct types of cell proliferation: one associated with non-antheridium cells and the other directly linked to antheridium initiation, differentiation, and the proliferation phase of spermatogenesis (Fig. 4, S2-S4). Beginning at 1 DAG, cells located in the apical region of the prothalli were not yet differentiated (orange arrowheads, Fig. 4A-B), and a few non-antheridium cells and their progeny continued to undergo one or two rounds of regular division before transitioning into the antheridium initiation program (Fig. 4A-J). These regular cell proliferation activities in the apical region (highlighted with orange arrowheads in Fig. 4A-C, F, J) align with the observations that male gametophytes, even under continuous antheridiogen exposure, continue to grow in size and add more non-antheridium cells during the first few days after germination (Fig. 1A, B).

In contrast, cell proliferation associated with antheridium development followed specific and conserved patterns (Figs. 4, S2-S4). In the presence of antheridiogen, male gametophytes begin initiating antheridia at an early stage when they consisted of only a few cells (Fig. 4A, S3A, S4A). Cells located in the basal region of the prothalli, particularly those near the spore coat, were the first to differentiate and initiate antheridia (as highlighted with dashed circles in Figs. 4A, S3A, S4A). As development progressed and most cells located in the basal region differentiated into antheridia, cells on either side of the apical region began to differentiate, continuing the orderly progression of antheridium formation (Fig. S2A-G). These live-imaged samples (Fig. 4, S2-S4) provided dynamic contexts for the static snapshots of male gametophytes observed at different DAGs in Figs.1 and 2.

**Fig. 5:**
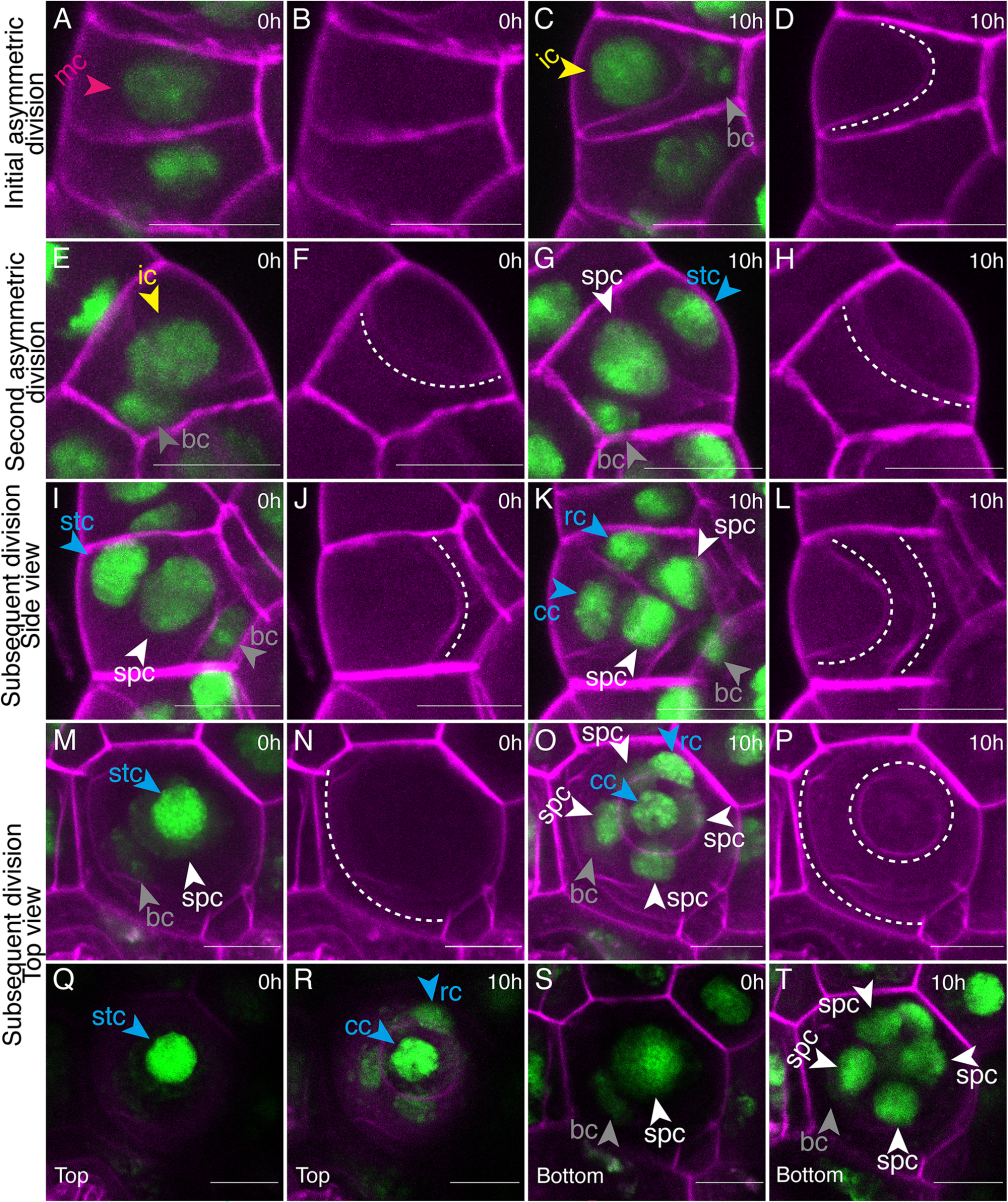
Confocal live imaging of cell division events associated with antheridium initiation and early development. **A**–**T** Confocal images showing nuclei (green) and cell outlines (magenta) during early antheridium development. Male gametophytes expressing the nuclear marker were transferred onto fresh CFM plates, stained with PI, rinsed with sterile water, and imaged by confocal microscopy at 0 h (**A**, **B**, **E**, **F**, **I**, **J**, **M**, **N**). After culturing for an additional 10 h on CFM in the growth chamber, the same male gametophytes were stained with PI again and imaged by confocal microscopy (**C**, **D**, **G**, **H**, **K**, **L**, **O**, **P**). **A**, **C**, **E**, **G**, **I**, **K**, **M**, **O** Merged images of GFP and PI channels. **B**, **D**, **F**, **H**, **J**, **L**, **N**, **P** PI channel showing cell outlines. **Q**–T Transverse sectional views taken from upper (top) and lower (bottom) positions along the z-axis of the antheridia shown in (M-P), visualizing individual nuclei in developing antheridia. Magenta arrowheads indicate antheridium mother cells (mc); yellow arrowheads indicate antheridium initial cells (ic); gray arrowheads indicate the basal shield cell (bc); blue arrowheads indicate sterile cells (stc), including ring cells (rc) and cap cells (cc), and white arrowheads indicate spermatogenous cells (spc). White dashed lines highlight newly formed cell walls in developing antheridia. Scale bars: 20 μm. At least three samples were examined at each indicated stage, showing comparable results.

We then examined each step of antheridium initiation and early development, together with the associated division patterns, based on time-lapse images collected over extended periods (Fig. 4, S2-S4). To complement these datasets and to visualize new cell wall formation, we performed an independent set of live imaging experiments in parallel, focusing on one division event at a time during early antheridium development (Fig. 5A-T). In these experiments, both GFP-labelled nuclei and PI-stained cell outlines were imaged at two different time points (Fig. 5A-T).

**Fig. 6:**
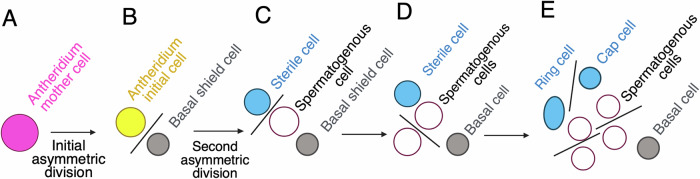
Diagrammatic illustration of antheridium initiation and differentiation. **A**, **B** The antheridium mother cell (magenta, **A** undergoes an initial asymmetric cell division, producing the antheridium initial cell (yellow) and basal shield cell (gray) (**B**). **B**, **C** The antheridium initial cell (yellow, **B**) divides again, giving rise to a spermatogenous cell (white) and a sterile cell (blue) (**C**). **C**, **D** The spermatogenous cell (white, **C**) divides to form two daughter spermatogenous cells (white) (**D**). **D**, **E** The sterile cell (blue) divides to form the cap (blue) and ring cell (blue), while spermatogenous cells (white) continue dividing, largely synchronously to form new spermatogenous cells (white). The basal shield cell remains undivided throughout antheridium development. This illustration was created in BioRender and modified in PowerPoint.

Antheridium development began from a single cell, the antheridium mother cell (magenta arrowhead, Fig. 4A). This cell and its progeny underwent successive divisions, ultimately forming a complex three-dimensional protrusion-like structure (two representative antheridia outlined by white dashed circles, Fig. 4A-P, S2A-F). In response to antheridiogen, the nucleus of the antheridium mother cell (indicated by the magenta arrowhead in Fig. 4A) underwent an initial asymmetric division, generating two daughter nuclei: one corresponding to the antheridium initial cell (yellow arrowhead, Fig. 4B) and the other to the basal or shield cell of the developing antheridium (gray arrowhead, Fig. 4B). Cell wall staining showed that this first division was associated with the formation of a newly curved cell wall (Fig. 5A-D). As summarized in Fig. 6A, B, this initial division step was highly conserved and was frequently observed in other antheridia from the same male gametophyte (yellow and gray arrowheads, Fig. 4A) and across different individuals (yellow and gray arrowheads in Figs. S3A-B and S4C).

The antheridium initial cell (yellow arrowhead, Fig. 4A) then underwent a second division, producing two nuclei: a larger, centrally localized spermatogenous nucleus (white arrowhead, Fig. 4B) and a smaller, peripherally positioned sterile nucleus (blue arrowhead, Fig. 4B), marking a conserved early developmental stage (Fig. 6B-C). Interestingly, the separation of the spermatogenous nucleus and the sterile nucleus was not directly accompanied by the formation of a new cell wall (Fig. 5E-H). The central spermatogenous nucleus subsequently divided, forming two spermatogenous nuclei positioned at the upper and lower regions of the antheridium along the Z-axis (white arrowheads, right circles in Fig. 4C-E; white arrowheads in Fig. 5I-L; summarized in Fig. 6C, D). These nuclei continued dividing, largely synchronously (white arrowheads, right circles in Fig. 4G-H and right circles in Fig. 4L-M; white arrowheads in Fig. 5M-T), eventually producing multiple spermatids within each antheridium (dashed circles, Fig. 4J-P, S2A-F). This pattern aligns with previously reports describing 32 spermatids per mature antheridium in Ceratopteris males^39^. Meanwhile, the sterile nucleus (blue arrowhead, Fig. 4E) divided once to give rise to the cap and ring cells (blue arrowheads in Fig. 4H-I), forming sterile tissue surrounding the spermatogenous cells. Cell wall staining revealed the formation of new curved walls associated with the development of the characteristic cap and ring structures of antheridia (blue arrowheads and dashed lines, Fig. 5I-P). This division pattern (summarized in Fig. 6D-E) was also consistently observed across independent antheridia, as indicated by blue arrowheads at 18 h (Fig. 4G) and 24 h (Fig. 4K-L), respectively. In contrast, the basal shield cell (gray arrowheads, Fig. 4A-B), once formed, remained undivided throughout the live imaging period (Figs. 4A-P, S2A-G). This observation was consistent with the results from snapshots of multiple antheridia at various DAGs, each showing a single basal shield cell per mature antheridium (Figs. 2A-F, S1C-D). Analysis of independent antheridia from three live-images male samples (Figs. 4, S2-S4) confirmed these conserved division patterns for the antheridium mother cell, antheridium initial cell, sterile cell, spermatogenous cell, and basal shield cell during antheridium initiation and early development (Fig. 6A-E).

### Lineage and division dynamics in male gametophytes

**Fig. 7:**
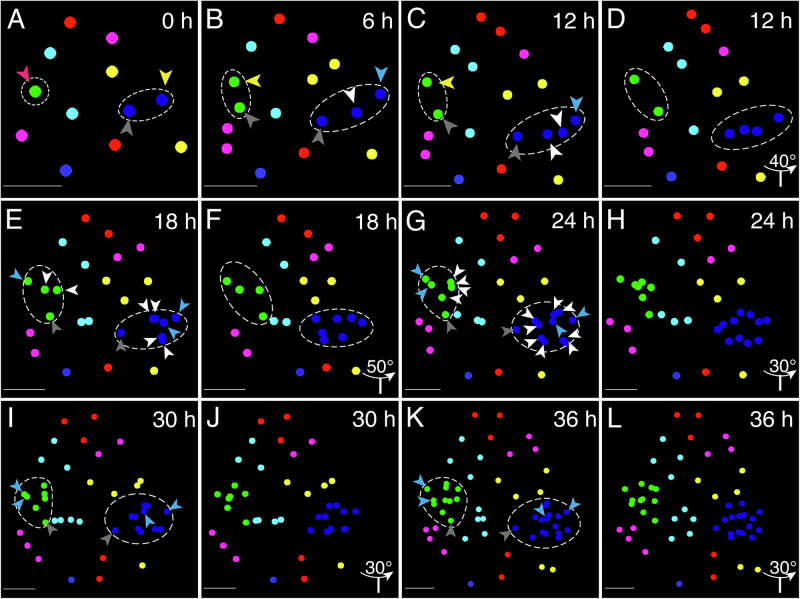
Cell lineage dynamics during antheridium development. **A**–**L** Cell lineage maps of the live-imaged male gametophyte (Sample 9, Fig. 4A-P). Nuclei in confocal images were segmented and assigned with unique IDs. Each dot represents the position of an individual nucleus. At 0 h, each nucleus was labeled with a different color relative to its neighboring nuclei as a reference for lineage analysis. At subsequent time points (6–36 h), progeny derived from the same nucleus are labeled with the same colors. **C**–**L** 3D rotational views of the lineage maps, shown from two angles along the Y-axis (angles indicated in each panel) for clear visualization of nuclear positions within developing antheridia from 12 h to 36 h. Scale bars (**A**–**L**): 50 μm. White dashed circles. **A**–**G**, **I**, **K** highlight two developing antheridia in the male gametophyte, as shown in Fig. 4. A magenta arrowhead indicates an antheridium mother cell that gives rise to subsequent antheridium development; yellow arrowheads indicate antheridium initial cells; gray arrowheads indicate basal cells; blue arrowheads indicate sterile cells, including cap and ring cells; and white arrowheads indicate spermatogenous cells. Three independent samples were analyzed, showing comparable results. Lineage maps for the other two samples are included in Fig. S5 and Fig. S6.

To quantitatively assess cellular dynamics and lineage progression during male gametophyte development, particularly antheridium initiation and proliferation, we performed 3D analyses of confocal time-lapse images (Supplementary Movies 1–7). GFP-labeled nuclei were identified from Z-stacks at each time point and assigned unique IDs, with their positions represented as color-coded solid circles. Nuclei and their progeny were identified and labelled across time points using consistent color coding to indicate lineage relationships; nuclei within the same antheridium were also color-matched for clarity (See methods for details). Using this approach, we constructed a dynamic atlas of Ceratopteris male gametophyte development at six-hour intervals, capturing progression from a single antheridium mother cell or an early two-nucleus stage to antheridia with the established basal shield cell, cap and ring cells, and multiple spermatogenous cells undergoing successive rounds of proliferation (Figs. 7, S5-S6).

**Fig. 8:**
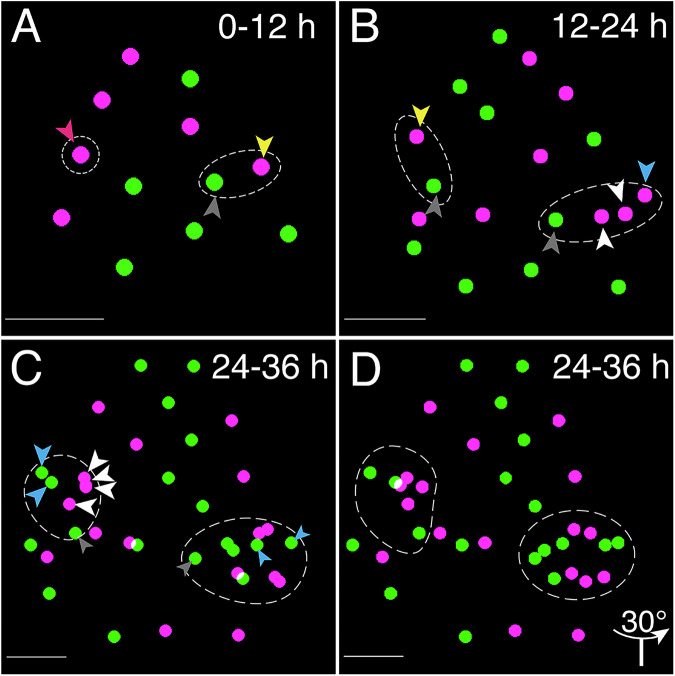
Cell division activity during antheridium development. **A**–**D** 3D projection views of cell division maps for successive 12-h intervals (0–12 h, 12–24 h, and 24–36 h). **C**–**D** 3D rotational views of the cell division map for 24–36 h interval, shown from two angles along the Y-axis (angles indicated in the panel) for clear visualization of nuclear positions within developing antheridia. Green dots represent nuclei that did not divide, while magenta dots represent nuclei that divided during the analyzed time frame. White dashed circles (**A**–**D**) highlight two developing antheridia in the male gametophyte, as shown in Fig. 4. A magenta arrowhead indicates an antheridium mother cell that gives rise to subsequent antheridium development; yellow arrowheads indicate antheridium initial cells; gray arrowheads indicate basal cells; blue arrowheads indicate sterile cells, including cap and ring cells; and white arrowheads indicate spermatogenous cells. Three independent samples were analyzed, showing comparable results. Division maps for the other two samples are included in Fig. S5 and Fig. S6.

Cell lineage maps revealed the developmental trajectories of individual antheridia from initiation through cell proliferation to sperm production (Fig. 7A-L). Using both 3D rotational views (Supplementary Movies 8-14) and Z-projection views (Fig. 7A-L) of reconstructed lineage maps, we visualized the spatial distribution and temporal progression of nuclei with distinct fates. In two representative antheridia, although the total number of nuclei increased over time, lineage contributions differed markedly. Following the initial separation of the antheridium initial nucleus (yellow arrowheads, Fig. 7A-B) from the basal shield nucleus (gray arrowheads, Fig. 7A-B), the basal nucleus remained undivided at the base of the antheridium (Fig. 7A-C, E, G, I, K). Subsequently, the spermatogenous nucleus (white arrowhead, Fig. 7B) separated from the sterile nucleus (blue arrowhead, Fig. 7B); the former underwent repeated divisions to generate centrally located spermatogenous nuclei, while the latter divided only once to form the surrounding cap and ring cells. We also generated cell division maps at 12-h intervals, highlighting divided cells in magenta and undivided cells in green (Fig. 8A-D, Supplementary Movies 15-17, Figs. S5L-N, S6L-N). These maps revealed distinct patterns of division activity in both non-antheridium cells and developing antheridia. During the early stage (0–12 h, Fig. 8A), most cells in the male gametophytes underwent division. In contrast, at later stages (12–24 h and 24–36 h; Fig. 8B-D), as development shifted from prothallus expansion to antheridium formation, division activity in non-antheridium cells progressively declined and eventually ceased, while nuclei within developing antheridia continued to proliferate. Within each antheridium, division activity varied among nuclei with different fates. Consistent with the lineage maps, the basal shield cell remained mitotically inactive throughout development, a consistent observation across all antheridia examined (Fig. 8A-D), the sterile nucleus divided only to generate the cap and ring cells, and the spermatogenous nuclei underwent sustained division to form multiple sperm cells (Fig. 8A-D).

### ABA rapidly suppresses antheridiogen-induced antheridium initiation and spermatogenous cell proliferation in developing antheridia

**Fig. 9:**
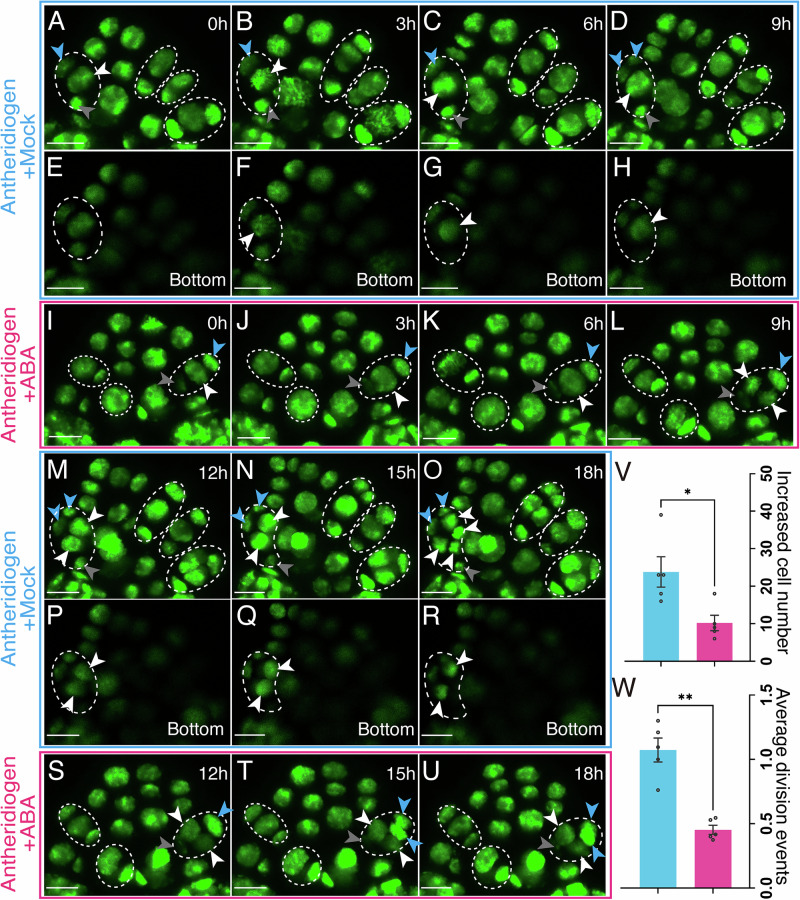
Time-lapse confocal imaging of antheridium development in the presence of antheridiogen alone and with both antheridiogen and ABA. **A**–**U** Z-projection and sectional views of male gametophytes expressing the *pCrUBQ10::H2B-GFP::3’CrUBQ10* nuclear reporter. **A**–**H**, **M**–**R** At 2 DAG, a male gametophyte was transferred from CFM to fresh CFM with mock treatment (0 h, **A**) and imaged by confocal microscopy every three hours from 0 h to 18 h. **A**–**D**, **M**–**O** Z-projection views; (E-H, P-R) transverse sectional views showing nuclear positions within developing antheridia. **I**–**L**, **S**–**U** At 2 DAG, a male gametophyte was transferred from CFM to CFM with 2.5 μM ABA (0 h, I) and imaged every three hours from 0 h to 18 h. (I-L, S-U) Z-projection views. White dashed circles (A–U) highlight developing antheridia. Gray arrowheads indicate basal shield cells; blue arrowheads indicate sterile cells, including cap and ring cells; and white arrowheads indicate spermatogenous cells. Scale bars (A-U): 20 μm. (V) Average increase in cell number per sample over the 18-h imaging period. (W) Average number of cell division events per cell over the same period. (V-W) Blue bars: samples grown in the presence of antheridiogen and mock treatment (*n* = 5 biologically independent samples); magenta bars: samples grown in the presence of both antheridiogen and ABA (*n* = 5 biologically independent samples). *, *p* < 0.05. **, *p* < 0.01 (Two-tailed Student’s *t*-test). (V) Bars and error bars represent mean ± SEM (mock treatment: 23.80 ± 4.04; ABA treatment: 10.20 ± 2.06). (W) Bars and error bars represent mean ± SEM (mock treatment: 1.07 ± 0.09; ABA treatment: 0.45 ± 0.04). Additional biological replicates are shown in Figs. S7 and S8, and detailed quantification of cell number and cell division events is shown in Figs. S9-S11. Source data for panels (V-W) are provided in Supplementary Data 1-2.

Previous studies have suggested that ABA antagonizes antheridiogen during male differentiation programming^38,42^. To uncover the cellular basis of this antagonism at high resolution, we performed a new set of time-lapse imaging experiments using the H2B-GFP nuclear marker (Figs. 9A-U, S7A-E1, S8A1-E1). Transgenic spores were initially cultured on CFM plates to induce male differentiation. At 2 DAG, male gametophytes were transferred to fresh CFM plates supplemented with either ABA or a mock treatment, and were imaged every three hours, focusing on developing antheridia containing no more than four nuclei (Fig. 9A-U, as illustrated in Fig. 6A-C). Consistent with earlier observations (Figs. 4, 7), antheridiogen alone promoted new antheridium initiation and continued cell division in developing antheridia. Under this condition, several antheridium initial cells or early spermatogenous cells were observed to divide within the first three hours (white dashed circles, Fig. 9A-B, E-F), and active proliferation continued in both spermatogenous and sterile lineages, resulting in antheridia (as highlighted in white dashed circles, Fig. 9A-H, M-R) with 5-9 nuclei by 18 hours. For instance, one representative antheridium began with one basal shield cell, one sterile cell, and one spermatogenous cell at 0 h (gray, white, and blue arrowheads, respectively, Fig. 9A) and developed into a structure containing 9 nuclei by 18 h (arrowheads, Fig. 9O, R). In contrast, when both antheridiogen and ABA were present, division activity in developing antheridia was remarkedly reduced. The number of nuclei in young antheridia (white dashed circles, Fig. 9I-L, S-U) increased only modestly, from one to two, or from two to three, over 9 hours (Fig. 9I-L), reaching only 3-5 nuclei by 18 hours (Fig. 9U). Particularly, one antheridium that initially contained three nuclei at 0 h (gray, white, blue arrowheads, Fig. 9I) only increased to 5 nuclei at 18 h (gray, white, blue arrowheads, Fig. 9U).

We then quantified cell division events in the apical region of male gametophytes, excluding mature or fully developed antheridia located in the basal region (see detailed analyses in Figs. S9-S11), over the 18-hour period. Five samples treated with antheridiogen alone (Figs. 9A-H, 9M-R, S7A1-E7) and five samples treated with both antheridiogen and ABA (Figs. 9I-L, 9S-U, S8A1-E7) were analyzed. By 18 hours, male gametophytes exposed to antheridiogen alone exhibited an average of 23.8 division events, corresponding to 1.07 divisions per nucleus (Fig. 9V, W, Supplementary Data 1-2). In contrast, male gametophytes exposed to both antheridiogen and ABA showed a significantly lower average of 10.2 division events, corresponding to 0.45 divisions per nucleus (Fig. 9V, W, Supplementary Data 1-2). Detailed antheridium cell division dynamics over time were further analyzed (Fig. S12 and Supplementary Data 3). Antheridia beginning with two nuclei at 0 h were clearly identified and analyzed at each time point when grown on CFM with mock treatment or ABA (Fig. S12, Supplementary Data 3). Compared with antheridiogen and mock treatment, antheridia exposed to both antheridiogen and ABA exhibited significantly reduced cell division activity at 15 h and 18 h, with an average 3.2 nuclei per antheridium under ABA treatment and 4.6 nuclei per antheridium under mock treatment at 18 h (Fig. S12, Supplementary Data 3). These quantitative results demonstrate that ABA substantially suppresses cell division associated with new antheridium formation and with developing antheridia during the early stages of male gametophyte development.

We next examined the effects of antheridiogen and ABA on sperm differentiation at late developmental stages, after completing spermatogenous cell proliferation (Fig. S13). At 3 DAG, male gametophytes expressing the nuclear marker were transferred from CFM to fresh FM (lacking antheridiogen) and supplemented with mock treatment, 2.5 μM ABA, or 10 μM ABA (Fig. S13A-F). Samples were imaged at 0 h and 24 h after treatment, focusing on antheridia that had completed or nearly completed spermatogenous cell proliferation prior to treatment. Time-lapse imaging showed that once spermatogenous cell division within antheridia was completed, these antheridia proceeded to undergo sperm differentiation even in the absence of antheridiogen. This process was characterized by the transition of solid nuclei turning into coiled, ring-like nuclear structures (Fig. S13A-B). Treatment with 2.5 μM ABA did not noticeably inhibit or delay this step (Fig. S13A-D), with antheridia displaying morphology comparable to mock-treated controls at 24 h. In contrast, in the presence of a higher concentration of ABA (10 μM), some antheridia were still able to undergo sperm differentiation program, but this process was delayed (Fig. S13A-B, E-F). These results indicate that once spermatogenous cell proliferation is completed, the sperm differentiation program proceeds independently of antheridiogen signaling. ABA appears to play a more prominent role during earlier stages of antheridium development, particularly in regulating antheridium initiation and spermatogenous cell proliferation, with a concentration dependent effect on the timing of sperm differentiation.

### Dynamic lineage alterations and division activity of male gametophytes in response to antheridiogen and ABA

**Fig. 10:**
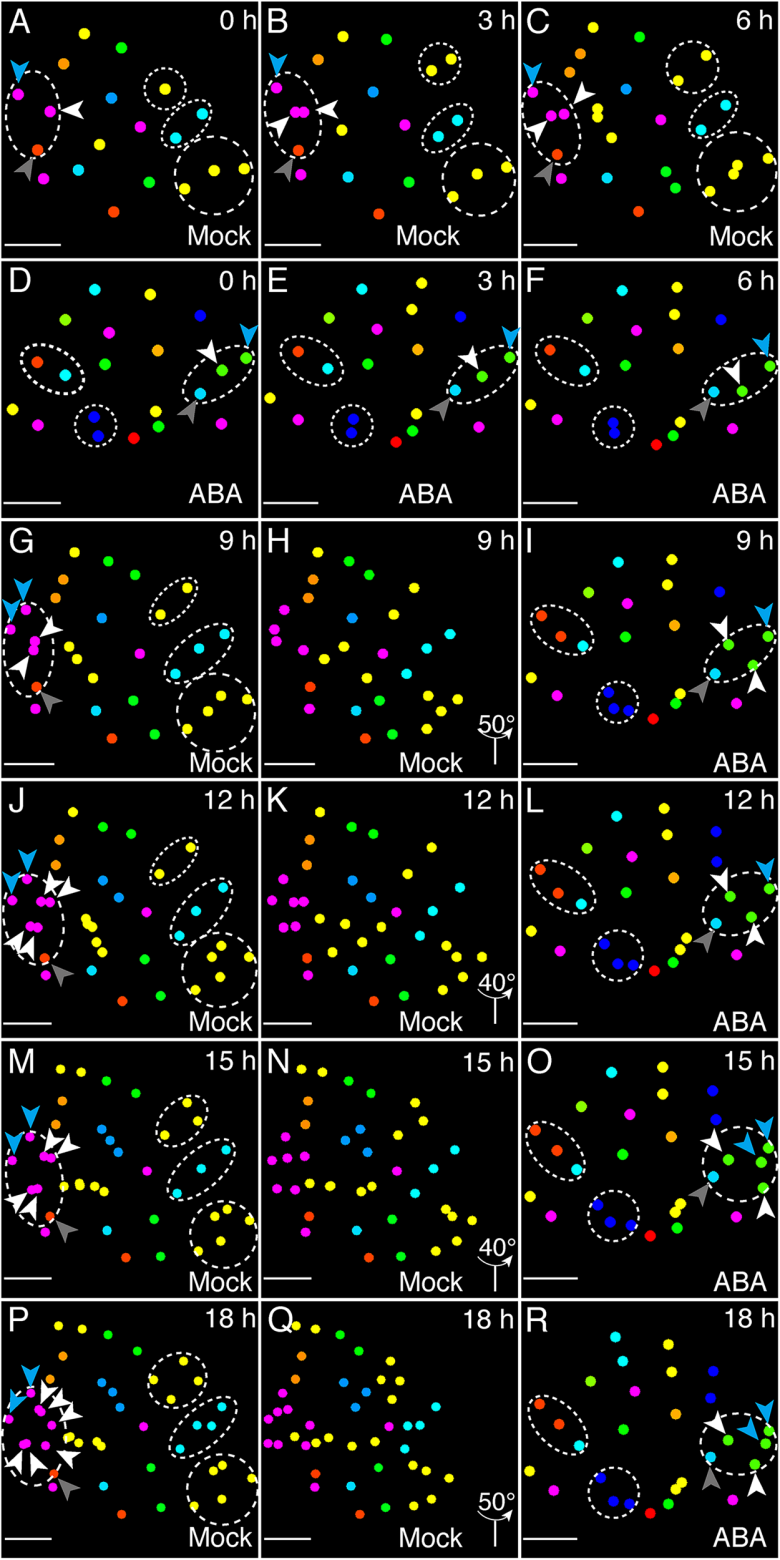
Cell lineage dynamics during antheridium development in the presence of antheridiogen and with both antheridiogen and ABA. **A**–**R** Cell lineage maps of the live-imaged male gametophytes (Fig. 9A-N). Nuclei in the confocal images were detected and labeled with unique IDs. Each dot represents the location of an individual nucleus. At 0 h, each nucleus was labeled with a different color than its neighboring nuclei as a reference for lineage analysis. At subsequent time points (3 h to 18 h), progeny from the same nucleus were labeled with the same colors. **A**–**C**, **G**, **H**, **J**, **K**, **M**, **N**, **P**–**Q** Cell lineage maps of the male gametophyte grown with antheridiogen only. **D**–**F**, **I**, **L**, **O**, **R** Cell lineage maps of the male gametophyte grown with both antheridiogen and ABA. **G**, **H**, **J**, **K**, **M**, **N**, **P**, **Q** 3D rotational views of the lineage maps from two angles along the Y-axis (as indicated in each panel) for clear visualization of the nuclei in developing antheridia from 9 h to 18 h. White dashed circles highlight several developing antheridia, as shown in Fig. 9A-N. Blue arrowheads, sterile cells including the cap and ring cells; white arrowheads, spermatogenous cells; gray arrowheads, the basal shield cell. Scale bars (**A**–**R**): 20 μm.

**Fig. 11:**
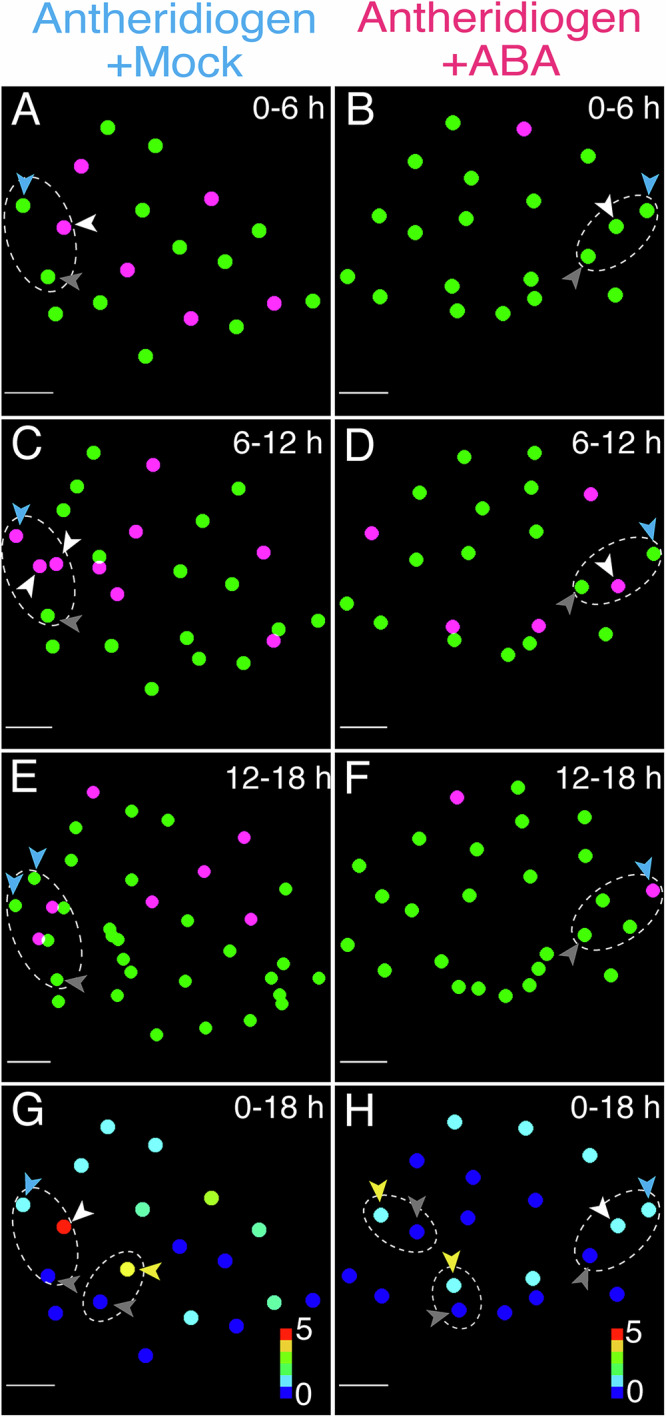
Cell division activity during antheridium development in the presence of antheridiogen alone and with both antheridiogen and ABA. **A**–**F** 3D-projection views of cell division maps for the male gametophytes shown in Fig. 9A-N, during each 6 h time frame (0–6 h, 6–12 h, and 12–18 h). Green dots represent nuclei that did not divide, while magenta dots represent nuclei that divided during each analyzed time frame. **G**, **H** 3D-projection views showing the quantitative division events over the 18 h period, with a color scale from red (maximum, 5 divisions) to blue (minimum, 0 divisions). Yellow arrowheads, the antheridium initial cell; gray arrowheads, the basal shield cell; blue arrowheads, sterile cells including the cap and ring cells; white arrowheads, spermatogenous cells. Scale bars: 20 μm.

We next focused on early antheridium development and examined cell lineage dynamics together with the spatial and temporal patterns of division activity in developing antheridia exposed to antheridiogen and ABA, using the same computational pipeline and three-dimensional analyses (Figs. 10 and 11). Based on time-lapse imaging data (Fig. 9), we constructed lineage maps in which each nucleus was represented by a color-coded solid circle indicating its position at each time point (Fig. 10A-R). This approach enabled clear visualization of lineage progression and fate relationships within developing antheridia, revealing how distinct cell lineages are established and altered at early developmental stages in response to antheridiogen and ABA over time.

In male gametophytes treated with antheridiogen alone, nuclei within developing antheridia (white dashed circles, Fig. 10A-C) continued to divide during the first six hours. In contrast, no nuclear division was observed in developing antheridia exposed to both antheridiogen and ABA during the same period (Fig. 10D-F). By 18 hours, antheridia in male gametophytes treated with antheridiogen alone generated more nuclei than those treated with both antheridiogen and ABA (Fig. 10P and R). To further examine this effect, we quantified division events at six-hour intervals and generated corresponding division maps (Fig. 11A-F). During the 0–6 h and 12-18 h intervals, gametophytes exposed to both antheridiogen and ABA exhibited only one or two nuclear divisions, while those exposed to antheridiogen alone showed six or seven divisions at each interval (Fig. 11A-B, E-F). Furthermore, a cumulative division atlas over the 18-h period revealed that multiple developing antheridia underwent 2-5 division events when exposed to antheridiogen alone, whereas none exceeded a single division event in the presence of both antheridiogen and ABA (Fig. 11G-H). For instance, one antheridium initial cell lineage underwent four divisions in the presence of antheridiogen (yellow arrowhead, Fig. 11G) but only one division in the presence of both ABA and antheridiogen (yellow arrowheads, Fig. 11H). Together, these results illustrate that ABA greatly suppresses antheridiogen-induced antheridium initiation and antheridium cell proliferation.

To further assess the effect of ABA on antheridium initiation and differentiation in male gametophytes, we examined wild-type (WT) and *gaia1* gametophytes^44^ grown on CFM with either mock or ABA treatment using confocal imaging (Fig. S14). Spores were surface-sterilized, plated on CFM, and transferred to mock- or ABA-supplemented CFM plates immediately after germination (0 DAG). At 2 DAG, mock-treated WT gametophytes initiated and developed multiple antheridia, with one representative antheridium labelled by blue and gray arrowheads (Fig. S14A), while those treated with ABA failed to initiate any antheridia, displaying a sexually undetermined phenotype (Fig. S14B). These observations were consistent with previous reports^42^. In contrast, *gaia1* gametophytes developed antheridia comparably under both mock and ABA treatments, with representative antheridia labelled by blue and gray arrowheads (Fig. S14C, D), consistent with previous findings that this mutant is insensitive to ABA^44^. These results highlight the opposing roles of antheridiogen and ABA in controlling both antheridium cell specification and differentiation.

## Discussion

Our analysis of Ceratopteris male gametophyte development provides a cellular and lineage resolved framework for understanding antheridium initiation, proliferation, and differentiation. By combining time lapse confocal imaging with 3D image analysis, we were able to follow individual nuclei over time and uncover a highly ordered developmental program in which cell fate specification and division behavior are tightly coordinated (Figs. 4–5, 7–8). Our results reveal early segregation of sterile and spermatogenous lineages from a single antheridium initial cell (Fig. 6). This developmental program is consistent across independent samples and produces structurally comparable antheridia even when initiation occurs at different times and positions within the same gametophyte (Figs. 2–8, S1-S6). The stereotyped sequence of asymmetric divisions revealed by dynamic lineage and division maps suggests that fate specification in fern antheridia is tightly regulated and robust to local developmental context.

The sterile and spermatogenous lineages, together with their division patterns observed in Ceratopteris antheridia, share similarity with antheridium developmental processes described in other fern species, such as *Pityrogramma calomelanos*^23,41^, suggesting that this developmental logic is broadly conserved among fern taxa. Although fern antheridia and angiosperm pollen differ markedly in structure and morphology, comparison of these two sperm-bearing systems reveals both conserved and lineage-specific features of male gametogenesis across land plants. In angiosperms, each pollen grain contains two distinct nuclear fates, the generative nucleus and vegetative nucleus^4,7,8^. In Arabidopsis, the vegetative cell gives rise to the pollen tube, whereas the generative nucleus undergoes a single division to produce two sperm nuclei^4,7,8^. In this respect, the early asymmetric division that separates spermatogenous and sterile lineages in fern male gametophytes follows a logic similar to that operating in seed plants, indicating a conserved principle of male gametophyte development.

Despite this shared logic, fern antheridia exhibit clear lineage-specific developmental innovations. Unlike pollen, fern antheridia generate a multicellular sterile tissue composed of cap, ring, and basal shield cells that physically surround the spermatogenous core (Figs. 5, S1). Our data show that, different from the largely mitotically inactive vegetative lineage in pollen, the sterile lineage in fern antheridia undergoes additional but limited divisions to generate these specialized cell types (Figs. 5–8). This architecture likely reflects an adaptation to the free-living and water-dependent reproductive strategy of fern gametophytes. The presence of a multilayered sterile envelope may provide mechanical support and structure protection for spermatogenesis in small and exposed gametophytes growing in fluctuating environments, while controlled rupture of the cap cell enables precise timing of sperm release.

Differences between ferns and seed plants are also evident in the proliferative behavior of the spermatogenous lineage. In fern antheridia, spermatogenous cells undergo multiple rounds of largely synchronized division (Figs. 3–4) until each antheridium contains thirty two spermatids^26,39^, which then differentiate into motile sperm with flagella^12,46^. This contrasts sharply with pollen development, where the generative lineage undergoes divides only once to produce two sperm nuclei for double fertilization^4,7,8^. The fern strategy likely enhances fertilization success in aqueous environments by producing multiple motile sperm per antheridium, highlighting how conserved cell fate specification can be coupled with lineage-specific proliferative outputs. Together, these findings suggest that fern antheridia retain features that may reflect ancestral states of male gametophyte development in land plants, combining early fate segregation with sustained proliferative capacity and specialized sterile support tissues.

In addition to lineage programs, our study reveals a central role for hormonal regulation in controlling antheridium development. Previous studies suggested that the phytohormone ABA antagonizes antheridiogen activity, with gametophytes exposed to both hormones developing few or no antheridia^42,47^. In this study, time-lapse imaging reveals that ABA has a rapid and sustained inhibitory effect on antheridium initiation and cell proliferation during Ceratopteris male gametophyte development. Specifically, ABA disrupts antheridiogen-driven antheridium cell fate specification, resulting in nearly complete suppression of antheridium initiation at early stages (Fig. S14). Following antheridium fate determination, ABA substantially reduces spermatogenous cell division (Figs. 9–11, S12, S15, Supplementary Data 3–4), resulting in delayed antheridium maturation. These effects are consistent with recent work showing that ABA inhibits cell proliferation in the meristem progenitor cell (MPC) lineage during male to hermaphrodite conversion in Ceratopteris^48^, suggesting that the inhibition of cell proliferation represents a general role of ABA across gametophyte sexual contexts. Additionally, once spermatogenous cell proliferation is completed, ABA displays a concentration-dependent effect on sperm differentiation, delaying but not completely blocking this process (Fig. S13). This observation raises the possibility that ABA influences sperm differentiation either directly or indirectly through its broader inhibitory effects on cellular growth programs^49,50^. Our data indicate that ABA-mediated suppression of both proliferative and maturation phases of spermatogenous cells are dependent on SnRK2-mediated signaling (Figs. S14, S15, Supplementary Data 4). In angiosperms, SnRK2 kinases are known to regulate cell cycle progression, chromatin status, and differentiation by modulating transcription factors and cell-cycle inhibitors^51–53^. ABA also suppresses cell proliferation and stabilizes non-dividing states in diverse developmental contexts, including embryos, endosperm, and root meristems in angiosperms^49,54,55^. Similar inhibitory effects on cell division have been reported in maize endosperms and in early embryo cells during coffee seed germination^54,55^. Additionally, ABA suppresses cell differentiation in Arabidopsis root meristems^49^.

Taken together, our findings establish a dynamic and quantitative framework for understanding male gametophyte development in ferns and highlight how conserved developmental logic is modulated by lineage-specific innovations and hormonal regulation. Future studies aimed at identifying hormone-responsive transcriptional targets, cell cycle regulators, and fate specification factors in fern antheridia will be essential for elucidating how hormonal signaling interfaces with lineage specific developmental programs during male gametogenesis, and for understanding the evolutionary conservation of reproductive regulatory mechanisms across land plants.

## Methods

### Plant materials and growth conditions

*Ceratopteris richardii* Hn-n^26^ was used as the wild type in this study. The *pCrUBQ10::H2B-GFP::3’CrUBQ10* transgenic nuclear marker line^45^ and the *gaia1* mutant^44^ have been reported previously. Spores were surface-sterilized and plated onto Petri dishes containing conditioned fern medium (CFM), which included antheridiogen to induce male differentiation. CFM was prepared as follows^38^. Ceratopteris *her19* spores^38^ were surface-sterilized and cultured in liquid FM (0.1 g /L) under continuous light with gentle shaking (110 rpm) for one month in a growth chamber. The liquid medium was then filtered, supplemented with 0.5x MS salts with vitamins (PhytoTechnology Laboratories), adjusted to pH 6.0, and solidified with 0.7% (w/v) agar (Sigma-Aldrich). Spores and germinated gametophytes were cultured under continuous light at 29 °C in growth chambers (Percival)^45^.

### Light microscope imaging

Spores of the Hn-n strain were surface-sterilized and spread on CFM to induce the male developmental program. After germination, male gametophytes were continuously exposed to antheridiogen. Light micrographs were captured using an Olympus CKX53 microscope equipped with an MLChrome 5 Pro digital camera, operated through the Mosaic2.3 software. Six male gametophytes were live-imaged at 2 DAG and 5 DAG, with one representative sample shown in Fig. 1A-B.

### Confocal microscope imaging

The spores of Hn-n were surface-sterilized and spread on CFM. Different male gametophytes at 3–6 DAG were randomly selected, stained with propidium iodide (PI) for 1 min in the dark, and rinsed three times with sterilized water. The samples (Fig. 1C, E, G, I) were then imaged using a Zeiss LSM880 upright confocal microscope to visualize cell outlines. After imaging, the same samples (Fig. 1D, F, H, J) were treated with 100% ethanol for 1 min, rinsed with sterilized water, and imaged again by confocal microscopy to visualize nuclei.

Spores of the *pCrUBQ10::H2B-GFP::3’CrUBQ10* reporter transgenic lines were also surface-sterilized and spread on CFM. To assess the antheridium developmental process, different male gametophytes at 3, 4, or 5 DAG were randomly selected, stained with PI for 1 min, and imaged by confocal microscopy. The Z-projection views of confocal image stacks (Fig. 2A-E, S1A-D) were generated using ImageJ/Fiji. Antheridia at late developmental stages, either before or during sperm differentiation (Fig. 3A-X), were stained with PI and imaged by confocal microscopy. Both Z-projection views and optical transverse section views were generated using ImageJ/Fiji to visualize nuclear organization and cellular architecture in antheridia.

To visualize cell division patterns during antheridium initiation and early development, live imaging was performed using a 10-hour interval (Fig. 5A-T), combined with PI staining to reveal cell outlines and newly formed cell walls. Spores of Ceratopteris transgenic lines expressing the nuclear marker *pCrUBQ10::H2BGFP::3’CrUBQ10* were surface-sterilized and plated on CFM. At 2 DAG, male gametophytes were randomly selected and stained with 1 mg/mL PI. After rinsing with sterile water, the gametophytes were transferred to fresh CFM plates and imaged using a LSM880 confocal microscope to visualize nuclei and cell outlines as the initial time point (0 h). The CFM plates were then returned to the growth chamber to maintain standard growth conditions and allow continued growth. After 10 h, the same male gametophytes were stained with PI again and imaged by confocal microscopy.

To continuously image male gametophytes exposed to antheridiogen (Fig. 4, S2-S4), non-invasive confocal time-lapse imaging^45,56^ was performed. Ceratopteris *pCrUBQ10::H2B-GFP::3’CrUBQ10* male gametophytes (at 2 DAG) were transferred from CFM to fresh CFM and imaged using confocal microscopy without mounting or staining to establish the initial time point (0 h). After imaging, the plates were returned to a growth chamber next to the confocal microscope and maintained under identical growth conditions. Live imaging of the nuclear marker was repeated every six hours until the early-formed antheridia reached maturity. At the last time point, the samples were stained with PI and imaged again to visualize cell outlines.

To capture division dynamics associated with antheridium initiation and spermatogenesis in the presence of antheridiogen alone or both antheridiogen and ABA (as shown in Figs. 9, S7-S8), male gametophytes of the Ceratopteris *pCrUBQ10::H2B-GFP::3’CrUBQ10* reporter at 2 DAG were transferred from CFM to CFM with mock treatment (0.1% ethanol, no ABA) or CFM with 2.5 μM ABA and 0.1% ethanol, and imaged by confocal microscopy as the first time point (0 h). The male gametophytes were then cultured under identical conditions in the growth chamber until the next time point. Live imaging was repeated every three hours during the 0–18 h time frame.

To assess the potential effects of ABA on sperm differentiation during late stages of antheridium development, spores of Ceratopteris lines expressing the nuclear marker were plated on CFM to promote male gametophyte development. At 3 DAG, male gametophytes with well-developed antheridia were randomly selected and transferred to FM with either mock treatment, 2.5 μM ABA, or 10 μM ABA. Male gametophytes were imaged by confocal microscopy immediately after the transfer as the initial time point (0 h) and then returned to the growth chamber under standard growth conditions. After 24 h, the same male gametophytes were imaged again by confocal microscopy to examine sperm differentiation within antheridia, as indicated by nuclei forming ring-like structures. More than three independent biological replicates were examined for each treatment, with consistent results observed across samples.

Spores of Ceratopteris WT and *gaia1* were surface-sterilized and plated on CFM. Once germinated, the gametophytes were transferred to CFM with either mock treatment (0.1% ethanol, no ABA) or CFM containing 2.5 μM ABA and 0.1% ethanol. After two days of treatment, samples were stained with PI for 1–2 min, rinsed with sterilized water, and imaged using confocal microscopy, as shown in Fig. S14. To compare cell division activity between WT and *gaia1* male gametophytes in response to ABA treatment (Fig. S15, Supplementary Data 4), spores of both lines were plated on CFM. At 3 DAG, male gametophytes from each genotype were randomly selected and divided into two groups, then transferred to either CFM supplemented with either mock or 2.5 μM ABA. After 18 h of treatment, gametophytes were treated with ethanol for 30 min, stained with PI, and imaged by confocal microscopy to visualize nuclei. Nucleus numbers were counted for non-antheridium cells and early-stage antheridia (each containing no more than seven nuclei) located in the apical half of the male gametophytes. The relative effects of ABA vs. mock treatment on cell proliferation were then calculated (Fig. S15, Supplementary Data 4).

All confocal imaging experiments were performed using a Zeiss LSM880 upright confocal microscope operated with ZEN Black software^37,45,56–59^. Gametophytes were imaged directly on Petri dishes using a Plan-Apochromat 10x/0.45 objective lens with 1.0 μm scanning intervals. For imaging GFP only, including samples expressing the H2B-GFP nuclear marker, excitation was performed using a 488 nm laser line, and emission was collected between 491 and 562 nm. DIC images were acquired via the transmission channel (T-PMT), using the same 488 nm laser line to visualize cell outlines. For imaging samples expressing the H2B-GFP nuclear marker with PI staining to visualize cell outlines, GFP excitation was performed using a 488 nm laser line and emission was collected between 491 and 535 nm. PI excitation staining was performed using a 561 nm laser line and emission was collected between 596 and 650 nm. For imaging PI-stained samples only, excitation was performed with a 561 nm laser line and emission was collected between 569 and 620 nm. Maximum-intensity Z projection views of the confocal stacks were processed using Fiji/Image J, except for samples shown in Figs. 1C-J and S14, where sum-intensity Z projection views of confocal stacks were generated. When necessary for clearer visualization of nuclei in gametophytes, the brightness of each entire confocal image was uniformly adjusted using Fiji/Image J.

### 3D nucleus identification

3D confocal images stacks of Ceratopteris *pCrUBQ10::H2B-GFP::3’CrUBQ10* reporter samples were first processed by manually identifying and annotating the central positions of nuclei using the editing tools in Fiji/ImageJ. A similar manual nucleus detection approach has previously been applied to identify nuclei in Arabidopsis root tissue^60^. Briefly, for image analysis, the grayscale intensity of each confocal image stack (8-bit format, maximum intensity value of 255) was uniformly reduced in Fiji/ImageJ to ensure that no pixel reached the maximum intensity level of 255. The central position of each nucleus was then manually identified and marked by setting the corresponding pixels to an intensity values of 255, thereby generating distinct high-intensity seed points relative to the rest of the image. Based on these manually annotated nucleus seed images, binary image stacks were automatically generated using a Matlab script titled “ManualNucleiDetection.m” (see Supplementary Data 5). In these binary images, pixels with an intensity value of 255 were assigned a value of 255, and all the other pixels with intensity values below 255 were set to 0. Unique labels were subsequently assigned to each annotated nucleus within the binary image stacks using the same Matlab script. Moreover, for visualization, spheres with a radius of 2 µm were plotted at each manually detected nucleus position to represent the nuclei, and each sphere was assigned a unique number corresponding to its nucleus label, also using the same Matlab script.

Two major types of errors can occur during the initial manual nucleus identification: incorrectly identified nuclei and missing nuclei. To address these issues, two Matlab scripts were developed (see Supplementary Data 5). The script, “BallNucleiDeletion.m,” was used to remove incorrectly identified nucleus labels from the detection results based on a manually generated list of their corresponding labels. The other script, “BallNucleiAddition.m” allowed for the addition of new 3D nuclei at manually specified x, y, z positions in cases where nuclei were missed during the manual identification. By integrating these manual error correction processes with the initial manual detection, highly accurate 3D nucleus detection in image stacks was achieved.

### 3D nuclei lineage reconstruction and cell division analysis

Three Matlab modules were developed for 3D nuclei lineage reconstruction and cell division analysis (see Supplementary Data 5).

Nuclear lineages over time were manually determined through visual examination and comparison of 3D nucleus detection images and the corresponding confocal z-stack images at each time point. The first module, “GenerateColorfulLineage.m,” used 3D nucleus detection results from multiple time points and manually annotated lineage data to reconstruct dynamic lineage images. Different colors were assigned to the founding nuclei at the initial time point. In following time points, nuclei descended from the same founding nuclei were assigned the same color, enabling lineage tracking. This lineage reconstruction module was used to generate images for multiple independent datasets, as shown in Figs. 7A-L, S5A-K, S6A-K, and 10A-R.

The second module, “NewCell_Divisions_ColorMap_3D_TwoColors.m,” generated two-color image stacks to visualize cell division dynamics. Using manually annotated nuclei lineage data, cell division events were automatically identified for specified time intervals. During the analyze periods, cells that underwent division were automatically colored magenta, while undivided cells were colored green. This two-color cell division visualization module was applied to generate images for multiple independent datasets, as shown in Fig. 8A-D, S5L-N, S6L-N, and 11A-F.

The third module, “NewCell_Divisions_ColorQuantification.m,” quantitatively represented the number of cell division events during specified time intervals using a six-color scale: blue indicates no cell division, cyan represents one cell division, green represents two divisions, yellow-green represents three divisions, yellow represents four divisions, and red represents five divisions. This quantitative division visualization module was used to generate the images shown in Fig. 11G-H.

### Cell division quantification and statistical analysis

The average increase in cell number and the average number of cell division events in male gametophytes in the presence of antheridiogen alone (CFM with mock treatment) or both antheridiogen and ABA (CFM with ABA) were quantified over the indicated analysis periods (Fig. 9V, W). At 0 h, nuclei in non-antheridium cells and in early-stage antheridia (with no more than four nuclei) were counted for each male gametophyte. The nuclei in these cells and antheridia were then counted again after 18 h of live imaging. Nucleus counts for individual samples at 0 h and 18 h are shown in Figs. S9–S11.

The increase in cell number for each sample was calculated as:

$${{{\rm{Increase}}}}\; {{{\rm{in}}}}\; {{{\rm{cell}}}}\; {{{\rm{number}}}}= \, \left({{{\rm{total}}}}\; {{{\rm{number}}}}\; {{{\rm{of}}}}\; {{{\rm{nuclei}}}}\; {{{\rm{at}}}}\,18{{{\rm{h}}}}\right)\\ - \, ({{{\rm{total}}}}\; {{{\rm{number}}}}\; {{{\rm{of}}}}\; {{{\rm{nuclei}}}}\; {{{\rm{at}}}}\,0{{{\rm{h}}}}).$$

The average increase in cell number was calculated from five independent biological samples for each treatment.

Cell division activity was expressed as the number of division events per cell, calculated as:

$${{{\rm{Cell}}}}\; {{{\rm{division}}}}\; {{{\rm{events}}}}\; {{{\rm{per}}}}\; {{{\rm{cell}}}}= 	 \, ({{{\rm{increase}}}}\; {{{\rm{in}}}}\; {{{\rm{cell}}}}\; {{{\rm{number}}}})/ \\ 	 ({{{\rm{total}}}}\; {{{\rm{number}}}}\; {{{\rm{of}}}}\; {{{\rm{nuclei}}}}\; {{{\rm{at}}}}\,0{{{\rm{h}}}}).$$

The average number of division events per cell was calculated from five independent biological samples for each treatment.

### Statistics and reproducibility

Statistical analyses were performed using Prism 9 based on independent biological replicates, with exact sample numbers indicated in the corresponding figure legends and Supplementary Data. Differences between mock and treatment groups were assessed using unpaired two- tailed Student’s *t* tests (Welch’s *t* test). Statistical significance was defined as *p* < 0.05, and exact *P* values are provided in Supplementary Data 1-4. For each experiment, at least three independent biological samples were included to ensure reproducibility.

## Supplementary information

Supplementary information

Description of Additional Supplementary Files

Supplementary Data 1

Supplementary Data 2

Supplementary Data 3

Supplementary Data 4

Supplementary Data 5

Supplementary Movie 1

Supplementary Movie 2

Supplementary Movie 3

Supplementary Movie 4

Supplementary Movie 5

Supplementary Movie 6

Supplementary Movie 7

Supplementary Movie 8

Supplementary Movie 9

Supplementary Movie 10

Supplementary Movie 11

Supplementary Movie 12

Supplementary Movie 13

Supplementary Movie 14

Supplementary Movie 15

Supplementary Movie 16

Supplementary Movie 17

## Data Availability

All data supporting the conclusions of this study are included in the main text, figures, Supplementary Figs., supplementary movies, or supplementary data. Source data underlying the graphs are provided in Supplementary Data 1-4.

## Code availability

The code used for image analysis is included in Supplementary Data 5 and is also available on Zenodo (10.5281/zenodo.19486941).
