# Supplementary material for: Cell division and lineage dynamics during antheridium differentiation and male gametophyte development in Ceratopteris richardii
Source: Commun Biol. 2026 Apr 30;9:911. doi: 10.1038/s42003-026-10135-w (PMC13338035; doi:10.1038/s42003-026-10135-w)
Supplement: Supplementary file 1 — Supplementary information [file 42003_2026_10135_MOESM1_ESM.pdf]

Supplementary information

Supplementary Figures

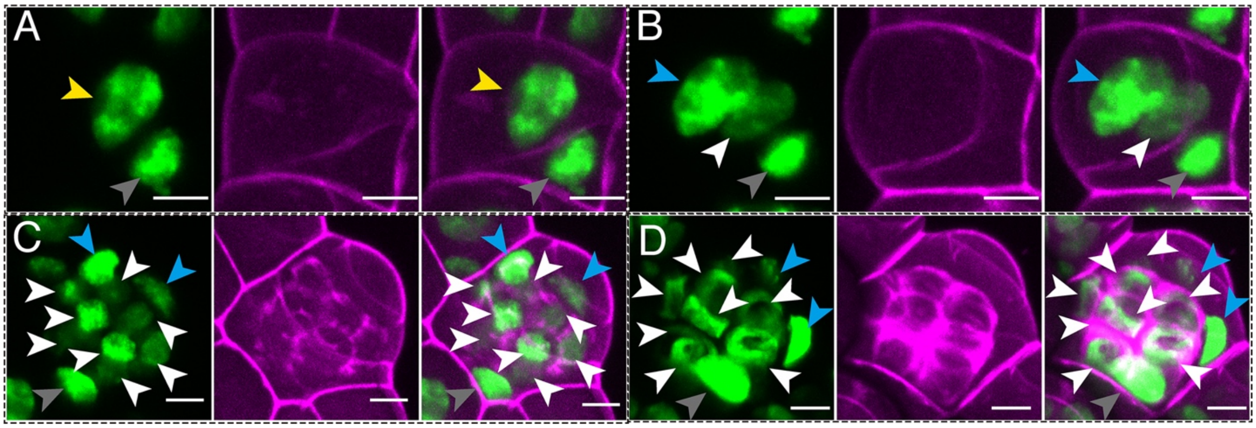

Supplementary Figure 1. Zoomed-in confocal views of antheridia at different developmental stages. (A-D) Enlarged Z-projection views of four antheridia at different developmental stages, as highlighted by white dashed circles in Fig. 2D and Fig. 2F. (A-D) Left, GFP channel (green); middle: PI counterstain (magenta); right: merged channels of GFP (green) and PI (magenta). Yellow arrowheads (A) indicate an antheridium initial cell; gray arrowheads (A-D) indicate basal shield cells; blue arrowheads (B-D) indicate sterile cells, including cap and ring cells; and white arrowheads (B-D) indicate spermatogenous cells. Scale bars: 10 µm.

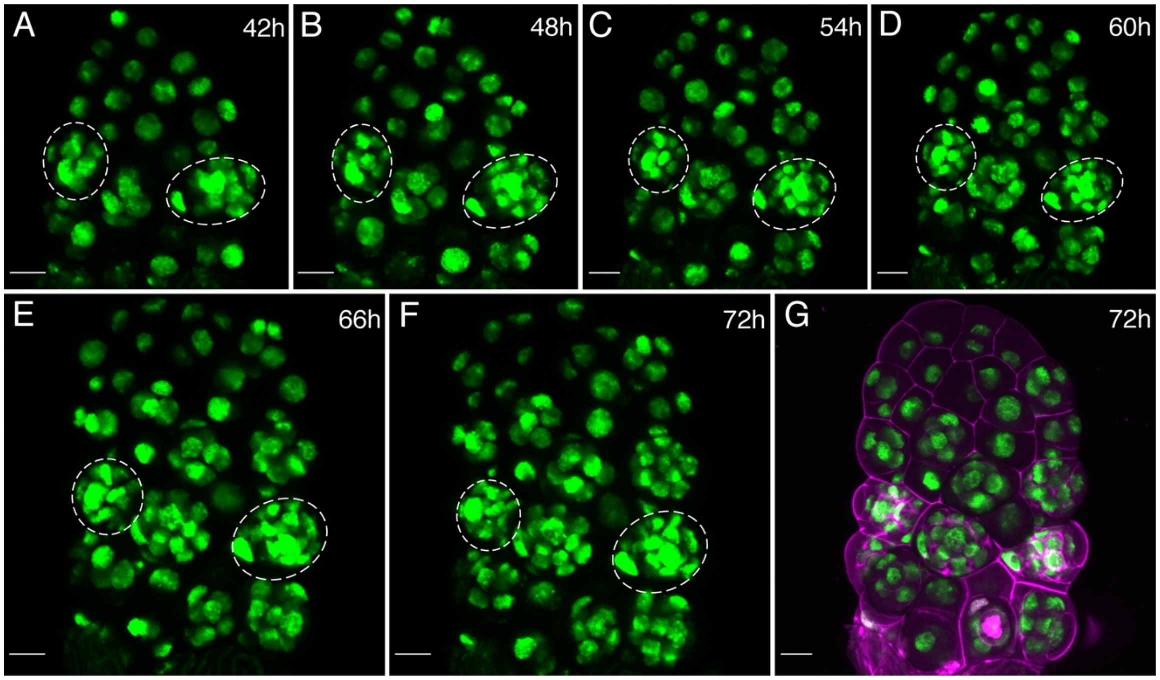

Supplementary Figure 2. Time-lapse confocal imaging of male gametophyte development in Sample 9 from 42 h to 72 h in response to antheridiogen. (A-G) Confocal images of a Ceratopteris male gametophyte (Sample 9) expressing the *pCrUBQ10::H2B-GFP::3'CrUBQ10* nuclear reporter. The gametophyte was grown on CFM and subsequently imaged every six hours. (A-G) Z-projection views of images from the 42-72 h time frames are shown here, and images from earlier time points (0-36 h) are present in Fig. 4. White dashed circles indicate developing antheridia. (A-F) GFP channel (green). (G) Merged channels of GFP and PI (showing cell outlines, magenta). Scale bars: 20 μm.

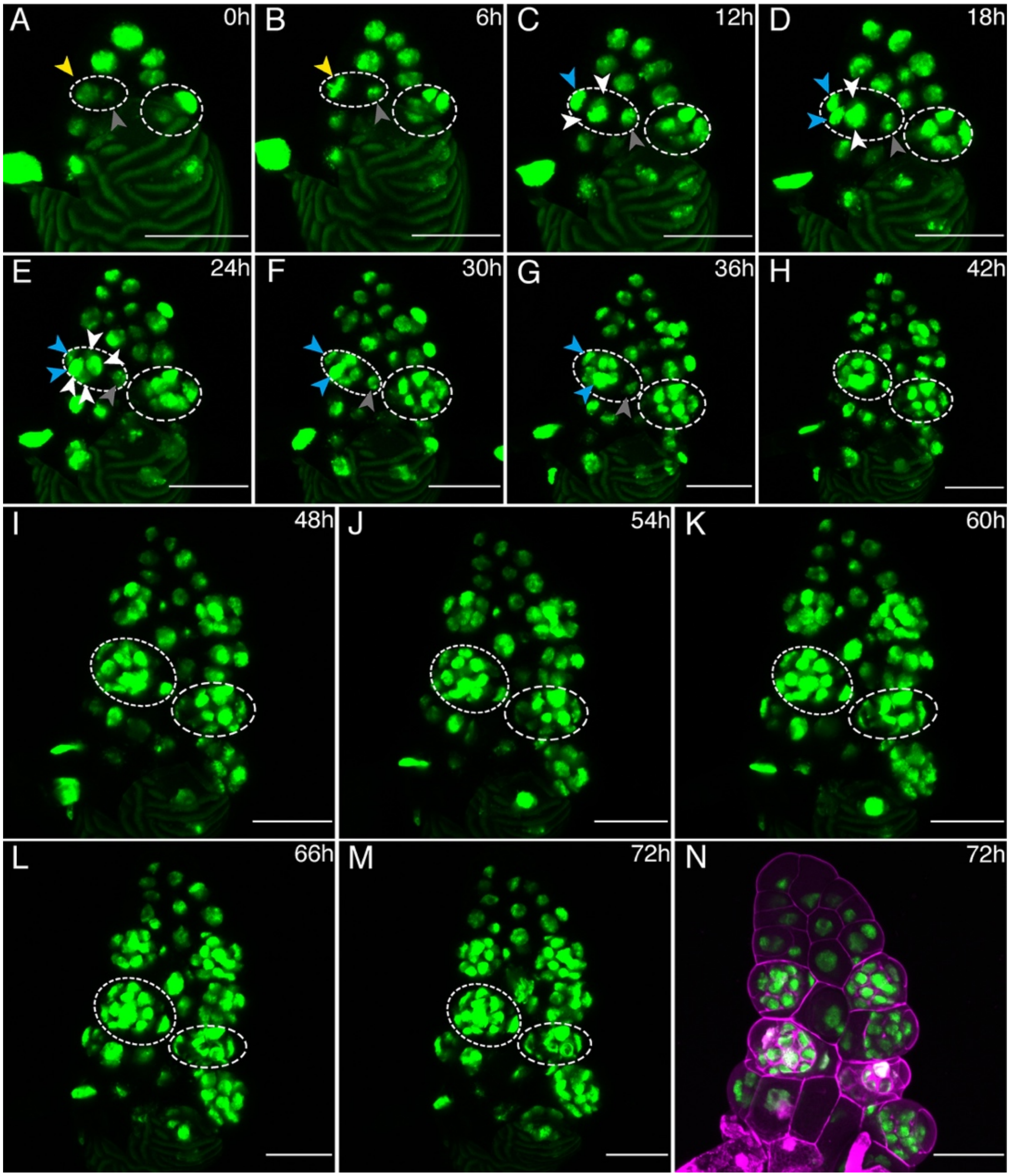

Supplementary Figure 3. Time-lapse confocal imaging of an independent sample (Sample 2) reveals cell dynamics during male development in response to antheridiogen. (A-N) Z-projection views of one Ceratopteris male gametophyte expressing the *pCrUBQ10::H2B-GFP::3'CrUBQ10* transgenic reporter. (A) At 1 DAG, the male gametophyte was live-imaged on CFM by laser scanning confocal microscopy as the first time point (0 hours, 0 h). (A-M) The gametophyte grown on CFM was imaged every six hours from 0 h (A) to 72 h (M), until several

antheridia matured. White dashed circles highlight the developmental process of two sperm-producing antheridia, from initiation to maturation (just before release sperms). (A-M) GFP channel (green) from 0-72 h; (N) merged GFP (green) and PI counterstain (magenta, showing cell outlines) at 72 h. Yellow arrowheads, the antheridium initial cell; gray arrowheads, the basal shield cell; blue arrowheads, sterile cells including the cap and ring cells; white arrowheads, spermatogenous cells. Scale bars (A-N): 50 μm. At least three samples were live-imaged under the same conditions and time frames, with comparable results. One representative sample (Sample 2) is shown here, with two independent replicates included in Fig. 4, Fig. S2, and Fig. S4.

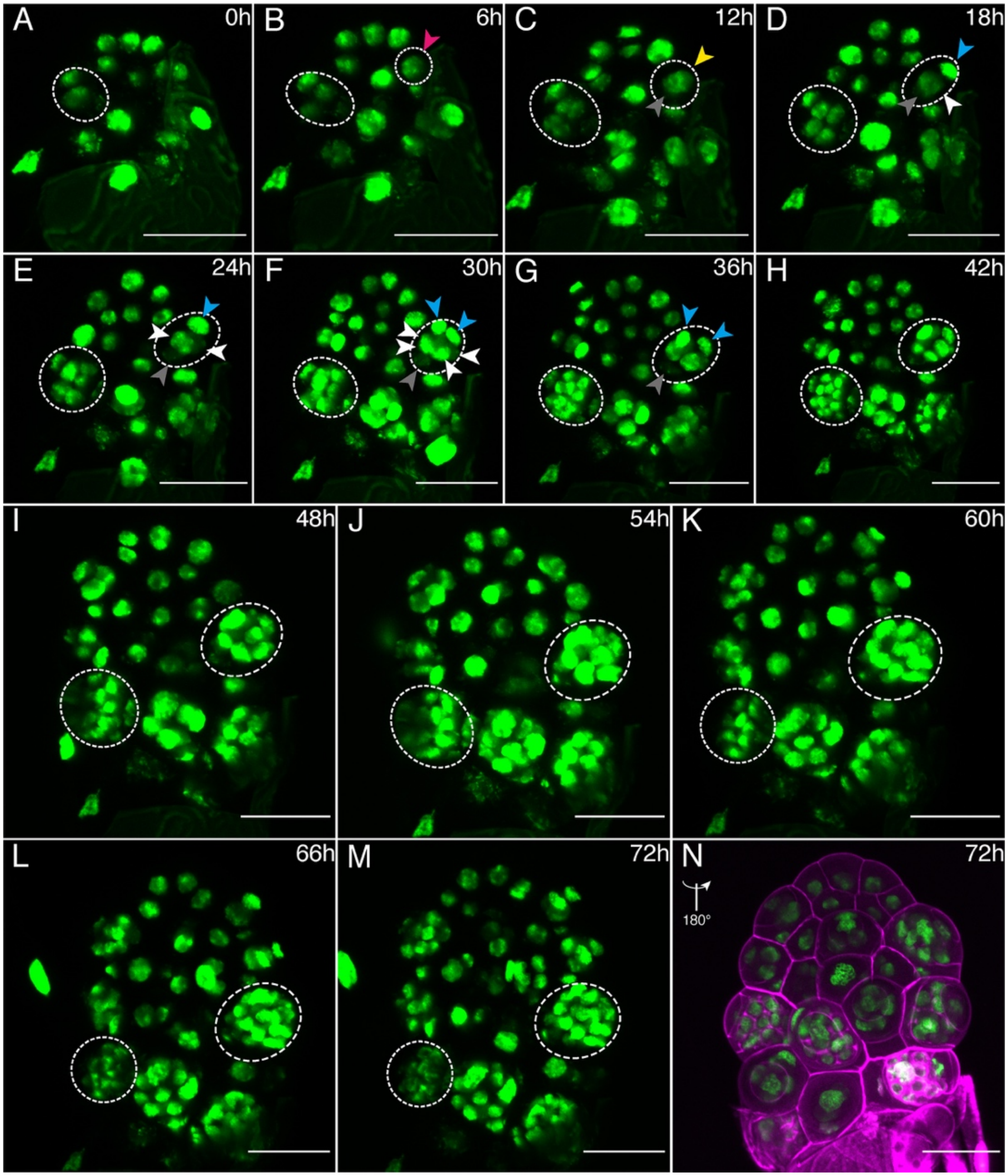

Supplementary Figure 4. Time-lapse confocal imaging of one independent sample (Sample 10) reveals cell dynamics during male development in response to antheridiogen. (A-N) Z-projection views of one Ceratopteris male gametophyte expressing the *pCrUBQ10::H2B-GFP::3'CrUBQ10* transgenic reporter. (A) At 1 DAG, the male gametophyte was live-imaged on CFM by laser scanning confocal microscopy as the first time point (0 hours, 0 h). (A-M) The gametophyte grown on CFM was imaged every six hours from 0 h (A) to 72 h (M), until several

antheridia matured. White dashed circles highlight the developmental process of two sperm-producing antheridia, from initiation to maturation (just before release sperms). (A-M) GFP channel (green) from 0-72 h; (N) merged GFP (green) and PI counterstain (magenta, showing cell outlines) at 72 h. Magenta arrowheads, the antheridium mother cell; yellow arrowheads, the antheridium initial cell; gray arrowheads, the basal shield cell; blue arrowheads, sterile cells including the cap and ring cells; white arrowheads, spermatogenous cells. Scale bars (A-N): 50 μm. At least three samples were live-imaged under the same conditions and time frames, with comparable results. One representative sample (Sample 10) is shown here, with two independent replicates included in Figs. 4, S2, and S3.

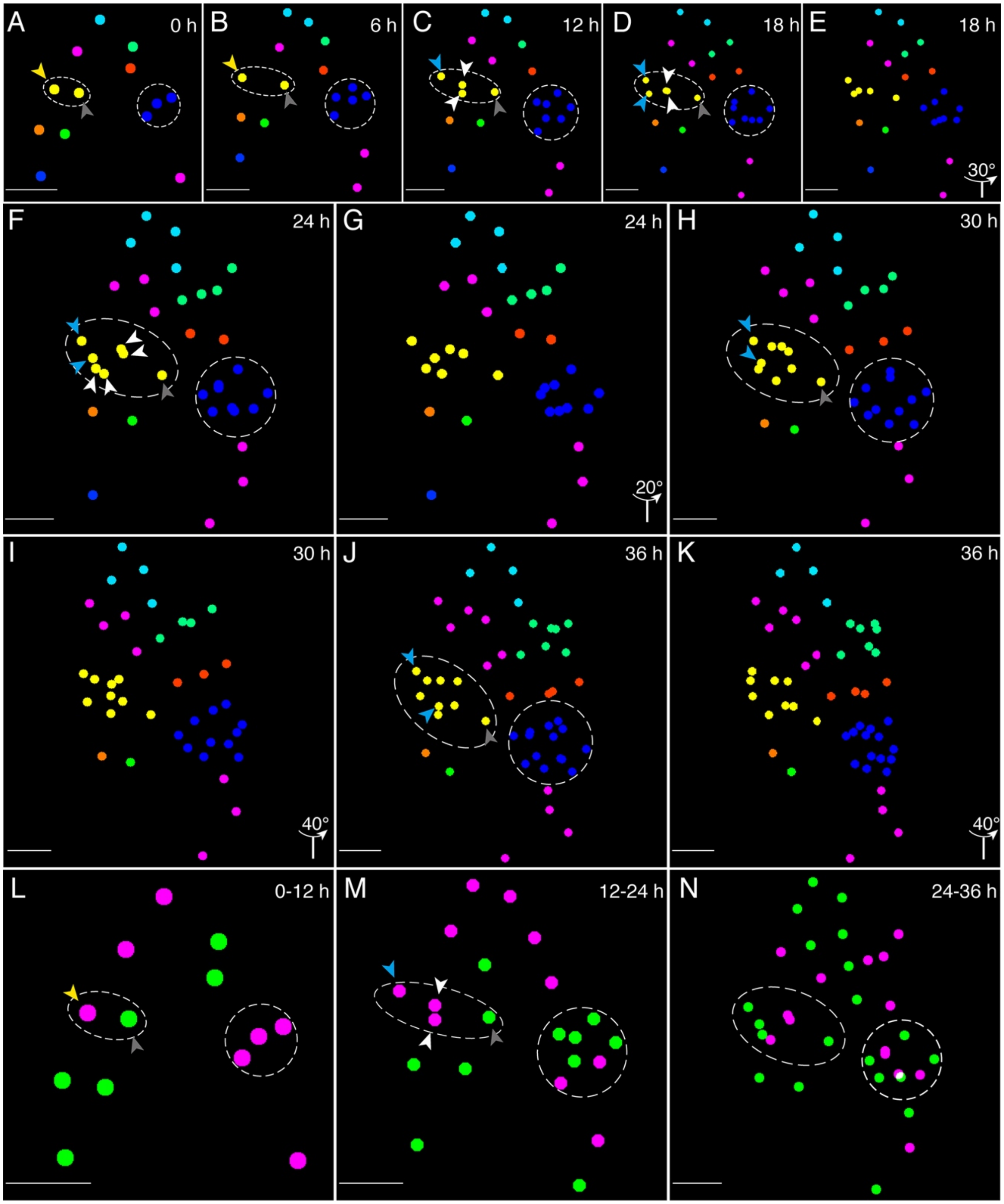

Supplementary Figure 5. Cell lineage dynamics and division activity of an independent sample (Sample 2) during male gametophyte development. (A-K) Cell lineage maps of the live-imaged male gametophyte (Sample 2, Fig. S3A-G). Nuclei in the confocal images were detected and labeled with unique IDs. Each dot represents the location of an individual nucleus. At 0 h, each nucleus was labeled with a different color than its neighboring nuclei as a reference for

lineage analysis. At subsequent time points (6 h to 36 h), progeny derived from the same nucleus were labeled with the same colors. (D-E, F-G, H-I, J-K) 3D rotational views of the lineage maps from two angles along the Y-axis (indicated in each panel) for clear visualization of the nuclei in developing antheridia from 18 h to 36 h. Scale bars (A-K): 20 μm. (L-N) 3D-projection views of cell division maps for each 12-h time frame (0-12 h, 12-24 h, and 24-36 h). Green dots represent nuclei that did not divide, while magenta dots represent nuclei that divided during the analyzed time frame. White dashed circles (A-D, F, H, J, L-N) highlight two developing antheridia in the male gametophyte, as shown in Fig. S3. Yellow arrowheads, the antheridium initial cell; gray arrowheads, the basal shield cell; blue arrowheads, sterile cells including the cap and ring cells; white arrowheads, spermatogenous cells. Three independent samples were analyzed, showing comparable results. Lineage and division maps of the other two samples are included in Figs. 7, 8, and S6, respectively.

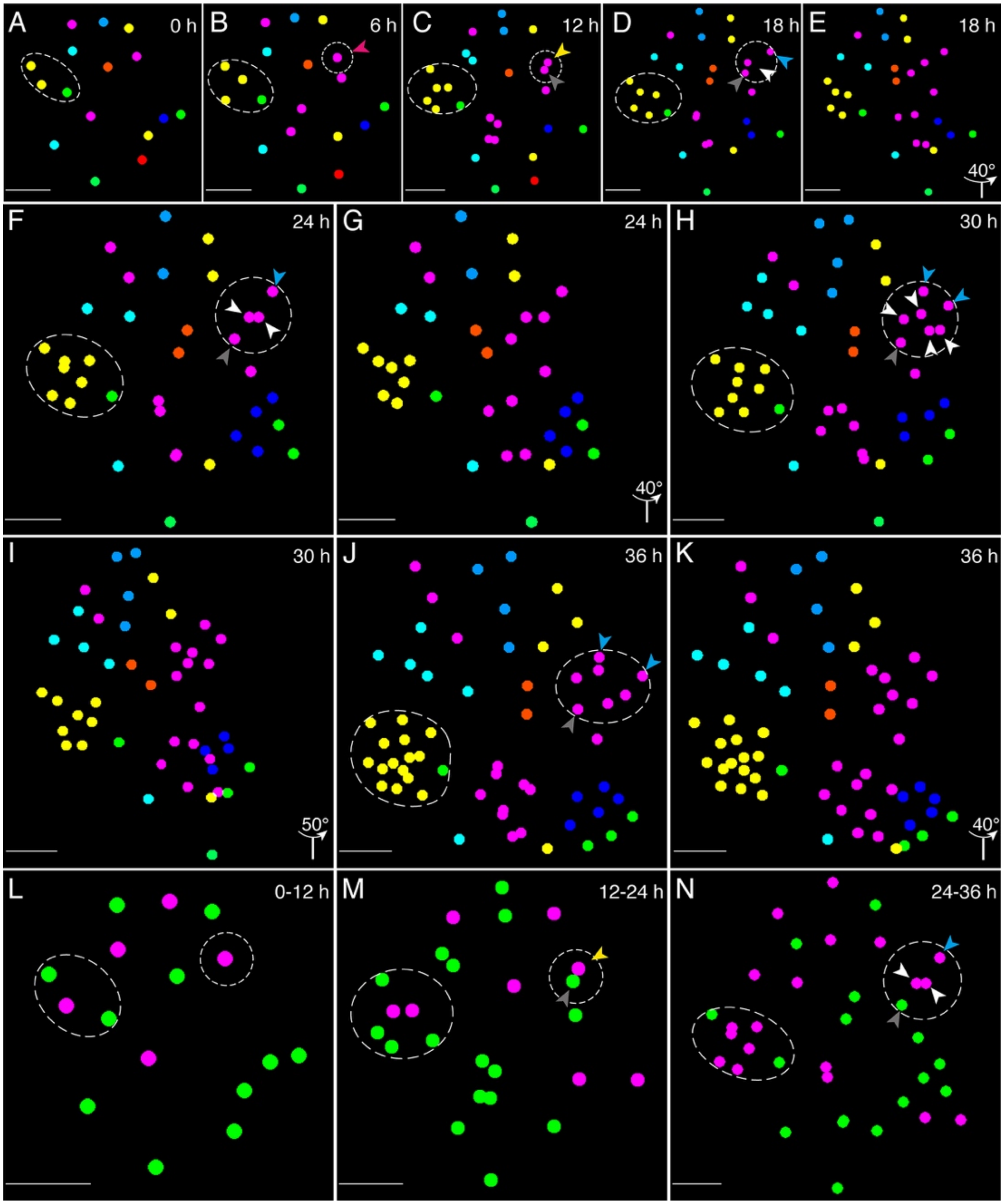

Supplementary Figure 6. Cell lineage dynamics and division activity of an independent sample (Sample 10) during male gametophyte development. (A-K) Cell lineage maps of the live-imaged male gametophyte (Sample 10, Fig. S4A-G). Nuclei in the confocal images were detected and labeled with unique IDs. Each dot represents the location of an individual nucleus. At 0 h, each nucleus was labeled with a different color than its neighboring nuclei as a reference for lineage analysis. At subsequent time points (6 h to 36 h), progeny derived from the same

nucleus were labeled with the same colors. (D-E, F-G, H-I, J-K) 3D rotational views of the lineage maps from two angles along the Y-axis (indicated in each panel) for clear visualization of the nuclei in developing antheridia from 18 h to 36 h. Scale bars (A-K): 20 μm. (L-N) 3D-projection views of cell division maps for each 12-h time frame (0-12 h, 12-24 h, and 24-36 h). Green dots represent nuclei that did not divide, while magenta dots represent nuclei that divided during the analyzed time frame. White dashed circles (A-F, H, J, L-N) highlight the two developing antheridia in the male gametophyte, as shown in Fig. S4. Magenta arrowheads, the antheridium mother cell; yellow arrowheads, the antheridium initial cell; gray arrowheads, the basal shield cell; blue arrowheads, sterile cells including the cap and ring cells; white arrowheads, spermatogenous cells. Three independent samples were analyzed, showing comparable results. Lineage and division maps of the other two samples are included in Figs. 7, 8, and S5, respectively.

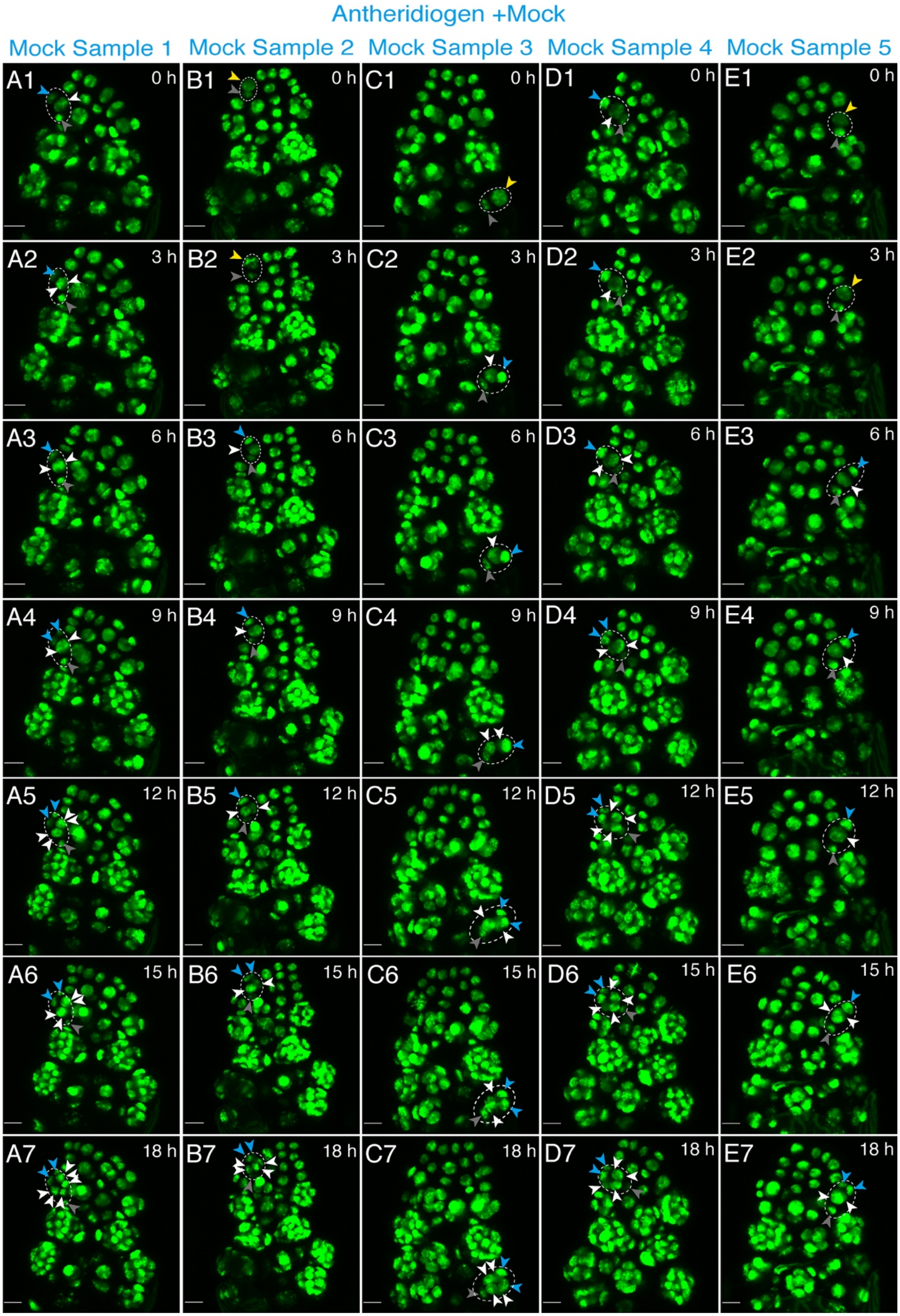

Antheridiogen +Mock
Mock Sample 1
Mock Sample 2
Mock Sample 3
Mock Sample 4
Mock Sample 5
A1
0 h
B1
0 h
C1
0 h
D1
0 h
E1
0 h
A2
3 h
B2
3 h
C2
3 h
D2
3 h
E2
3 h
A3
6 h
B3
6 h
C3
6 h
D3
6 h
E3
6 h
A4
9 h
B4
9 h
C4
9 h
D4
9 h
E4
9 h
A5
12 h
B5
12 h
C5
12 h
D5
12 h
E5
12 h
A6
15 h
B6
15 h
C6
15 h
D6
15 h
E6
15 h
A7
18 h
B7
18 h
C7
18 h
D7
18 h
E7
18 h

Supplementary Figure 7. Time-lapse confocal imaging of Ceratopteris male gametophytes grown in the presence of antheridiogen with mock treatment. (A1-E7) Z-projection views of five male gametophytes expressing the *pCrUBQ10::H2B-GFP::3'CrUBQ10* transgenic reporter. At 2 DAG, male gametophytes were transferred from CFM to fresh CFM with mock treatment and imaged by confocal microscopy every three hours from 0 h to 18 h. (A1-A7) Mock Sample 1, with zoomed-in images shown in Fig. 9; (B1-B7) Mock Sample 2; (C1-C7) Mock Sample 3; (D1-D7) Mock Sample 4; and (E1-E7) Mock Sample 5. White dashed circles (A1-E7) highlight a representative developing antheridium in each sample. Yellow arrowheads, antheridium initial cells; gray arrowheads, the basal shield cell; blue arrowheads, sterile cells including the cap and ring cells; white arrowheads, spermatogenous cells. Scale bars: 20 μm.

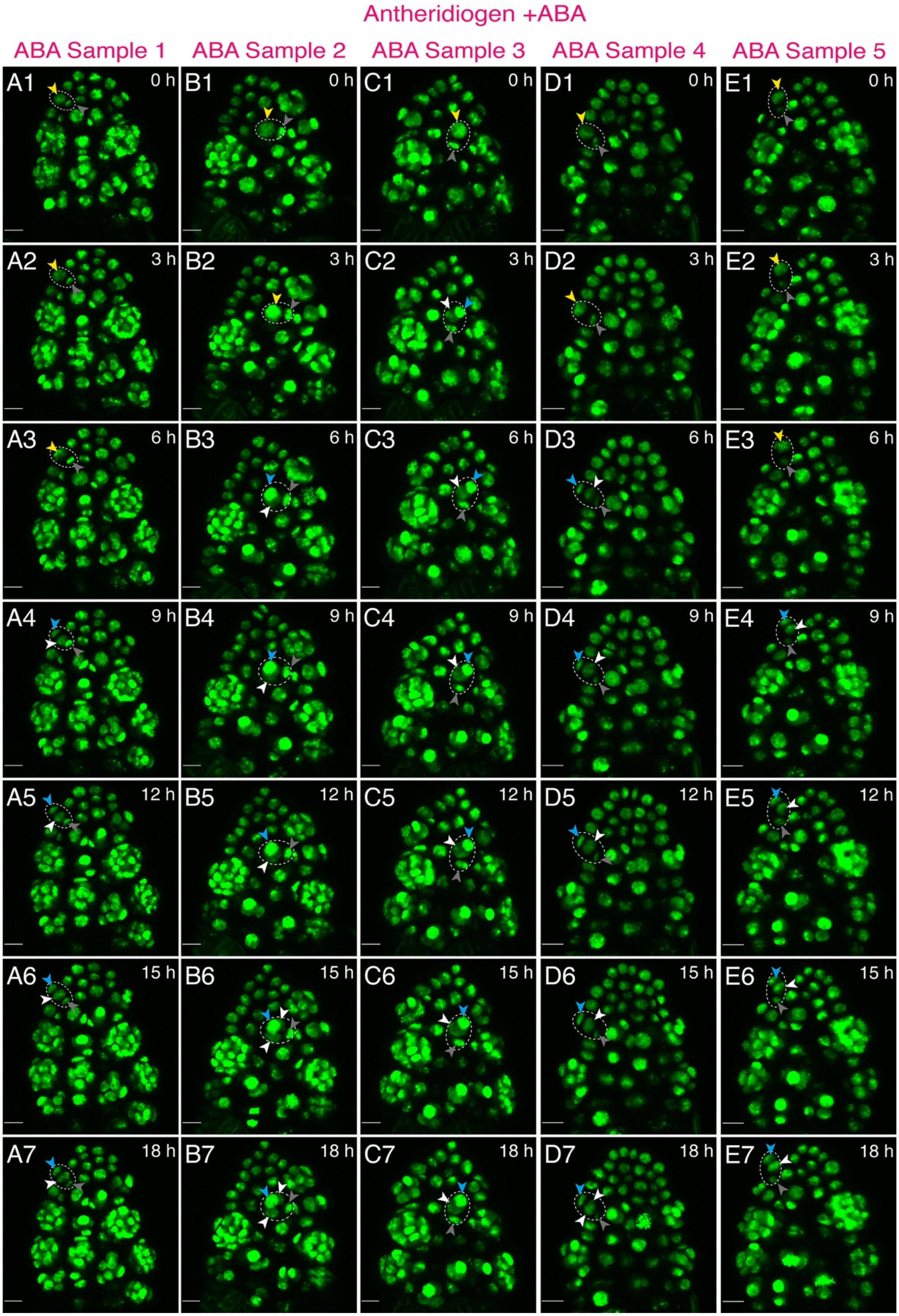

Antheridiogen +ABA
ABA Sample 1
ABA Sample 2
ABA Sample 3
ABA Sample 4
ABA Sample 5
A1 0 h
B1 0 h
C1 0 h
D1 0 h
E1 0 h
A2 3 h
B2 3 h
C2 3 h
D2 3 h
E2 3 h
A3 6 h
B3 6 h
C3 6 h
D3 6 h
E3 6 h
A4 9 h
B4 9 h
C4 9 h
D4 9 h
E4 9 h
A5 12 h
B5 12 h
C5 12 h
D5 12 h
E5 12 h
A6 15 h
B6 15 h
C6 15 h
D6 15 h
E6 15 h
A7 18 h
B7 18 h
C7 18 h
D7 18 h
E7 18 h

Supplementary Figure 8. Time-lapse confocal imaging of Ceratopteris male gametophytes in the presence of both antheridiogen and ABA. (A1-E7) Z-projection views of five male gametophytes expressing the *pCrUBQ10::H2B-GFP::3'CrUBQ10* transgenic reporter. At 2 DAG, male gametophytes were transferred from CFM to fresh CFM with 2.5 μM ABA and imaged by confocal microscopy every three hours from 0 h to 18 h. (A1-A7) ABA-treated Sample 1, with zoomed-in images shown in Fig. 9; (B1-B7) ABA-treated Sample 2; (C1-C7) ABA-treated Sample 3; (D1-D7) ABA-treated Sample 4; and (E1-E7) ABA-treated Sample 5. White dashed circles (A1-E7) highlight a representative developing antheridium in each sample. Yellow arrowheads; antheridium initial cells; gray arrowheads, the basal shield cell; blue arrowheads, sterile cells including cap and ring cells; white arrowheads, spermatogenous cells. Scale bars: 20 μm.

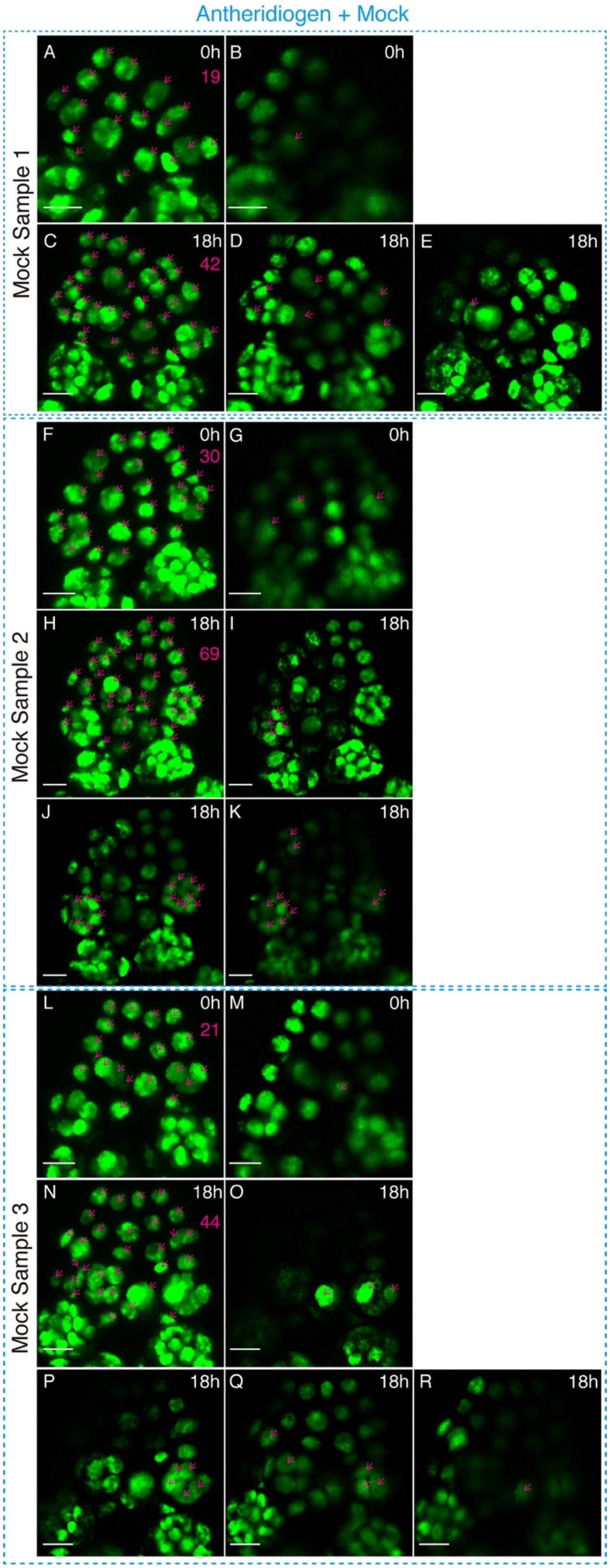

Antheridiogen + Mock
Mock Sample 1
Mock Sample 2
Mock Sample 3
A
0h
19
B
0h
C
18h
42
D
18h
E
18h
F
0h
30
G
0h
H
18h
69
I
18h
J
18h
K
18h
L
0h
21
M
0h
N
18h
44
O
18h
P
18h
Q
18h
R
18h

Supplementary Figure 9. Nucleus counts in Ceratopteris male gametophytes (Mock Samples 1-3) grown in the presence of antheridiogen with mock treatment over an 18-h interval. (A-R) Three male gametophyte samples were grown on CFM with mock treatment and imaged from 0 h to 18 h. (A-E) Z-projection views (A, C) and transverse sectional views (B, D, E) of Mock Sample 1 at 0 h and 18 h. (F-K) Z-projection views (F, H) and transverse sectional views (G, I-K) of Mock Sample 2 at 0 h and 18 h. (L-R) Z-projection views (L, N) and transverse sectional views (M, O-R) of Mock Sample 3 at 0 h and 18 h. Magenta arrows indicate nuclei analyzed for cell division activity. Magenta numbers indicate the total number of nuclei at 0 h and 18 h. Each nucleus is marked once by a magenta arrow in one of the confocal images. Scale bars: 20 μm.

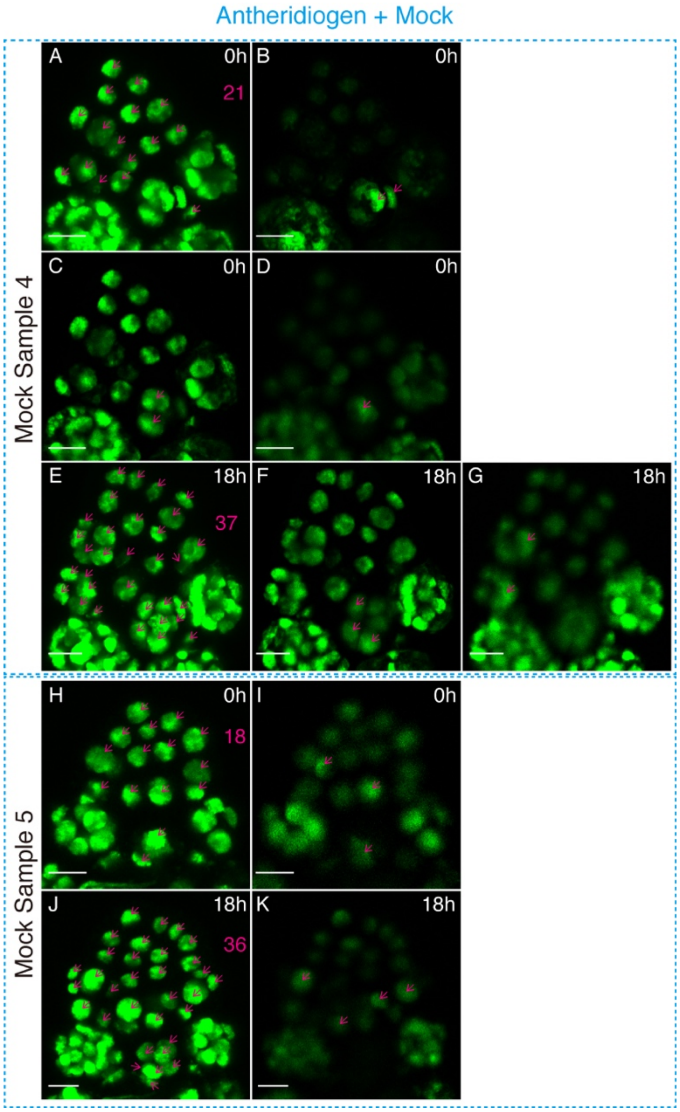

Supplementary Figure 10. Nucleus counts in Ceratopteris male gametophytes (Mock Samples 4-5) grown in the presence of antheridiogen with mock treatment over an 18-h interval. (A-K) Two male gametophyte samples were grown on CFM with mock treatment and imaged from 0 h to 18 h. (A-G) Z-projection views (A, E) and transverse sectional views (B-D, F-G) of Mock Sample 4 at 0 h and 18 h. (H-K) Z-projection views (H, J) and transverse sectional views (I, K) of Mock Sample 5 at 0 h and 18 h. Magenta arrows indicate nuclei analyzed for cell division activity. Magenta numbers indicate the total number of nuclei at 0 h and 18 h. Each nucleus is marked by a magenta arrow in one of the confocal images. Scale bars: 20 μm.

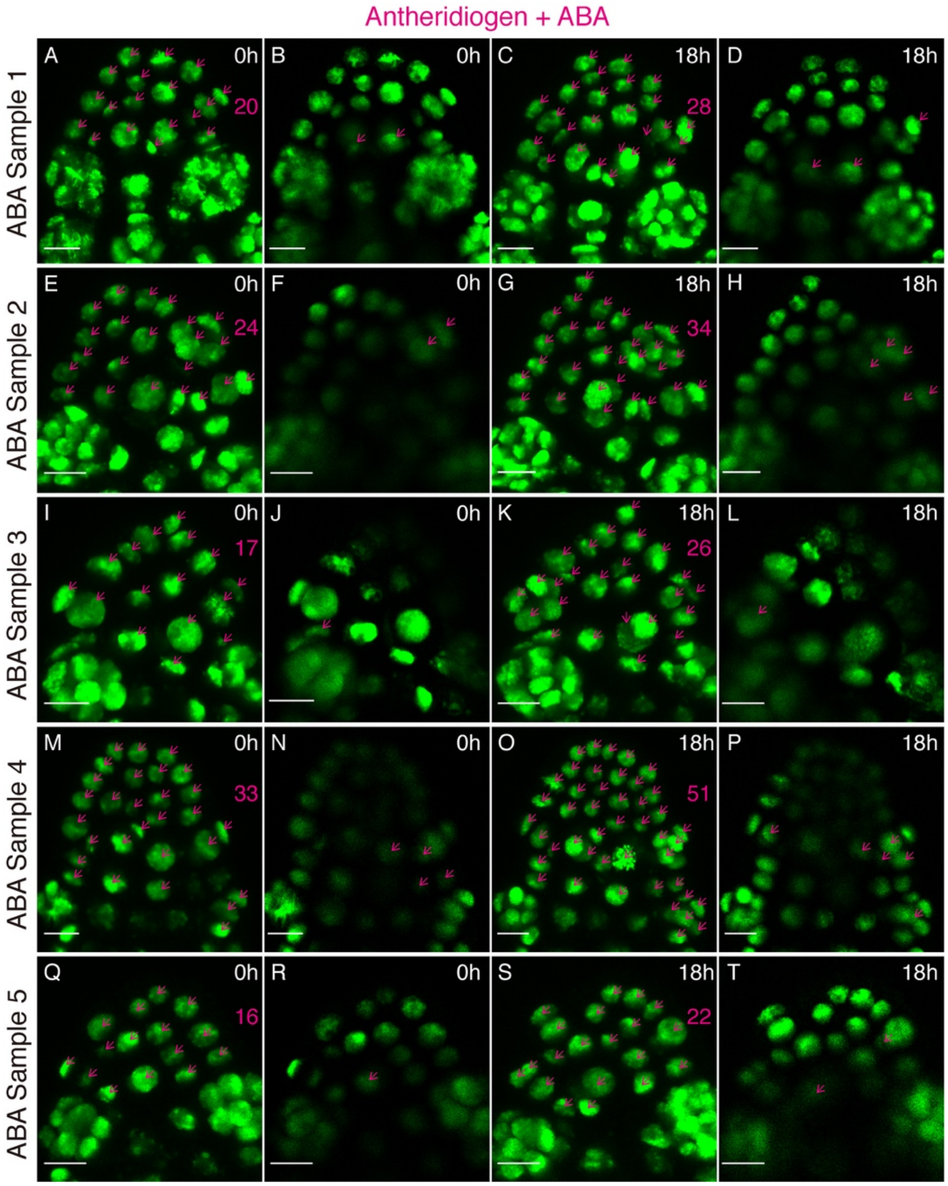

Supplementary Figure 11. Nucleus counts in Ceratopteris male gametophytes grown in the presence of antheridiogen with ABA treatment over an 18-h interval. (A-T) Five male gametophytes (ABA Samples 1-5) were grown on CFM with 2.5 μM ABA and imaged from 0 h to 18 h. Z-projection views (A, E, I, M, Q) and transverse sectional views (B, F, J, N, R) of individual male gametophytes at 0 h are shown. Z-projection views (C, G, K, O, S) and transverse sectional views (D, H, L, P, T) of the same gametophytes at 18 h after treatment are shown. Magenta arrows indicate nuclei analyzed for cell division activity. Magenta numbers indicate the total number of nuclei at 0 h and 18 h. Each nucleus is marked by a magenta arrow in one of the confocal images. Scale bars: 20 μm.

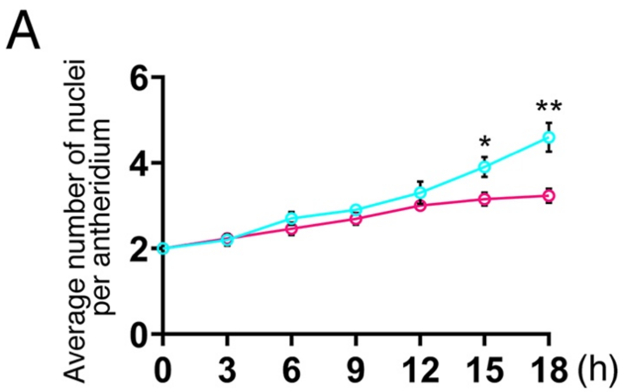

Supplementary Figure 12. Quantitative analysis of cell division activity during early antheridium development in response to antheridiogen with mock and ABA treatment. Antheridia at the two-nucleus developmental stage from male gametophytes shown in Figs. S7-S8 were analyzed every three hours over the 18-hour time period. The y-axis indicates the average number of nuclei per antherdium, and error bars represent the SEM. The x-axis indciates hours after treatment. Blue represents antheridia from male gametophyte grown on CFM with mock treatment. Magenta represents antheridia from male gametophtyes grown on CFM with 2.5 μM ABA. *, $p < 0.05$; **, $p < 0.01$; two-tailed Welch's *t*-test ($n = 10$ independent antheridia from 4 biologically independent gametophyte samples for mock treatment; $n = 13$ independent antheridia from 5 biologically independent gametophyte samples for ABA treatment). Source data are included in Supplementary Data 3.

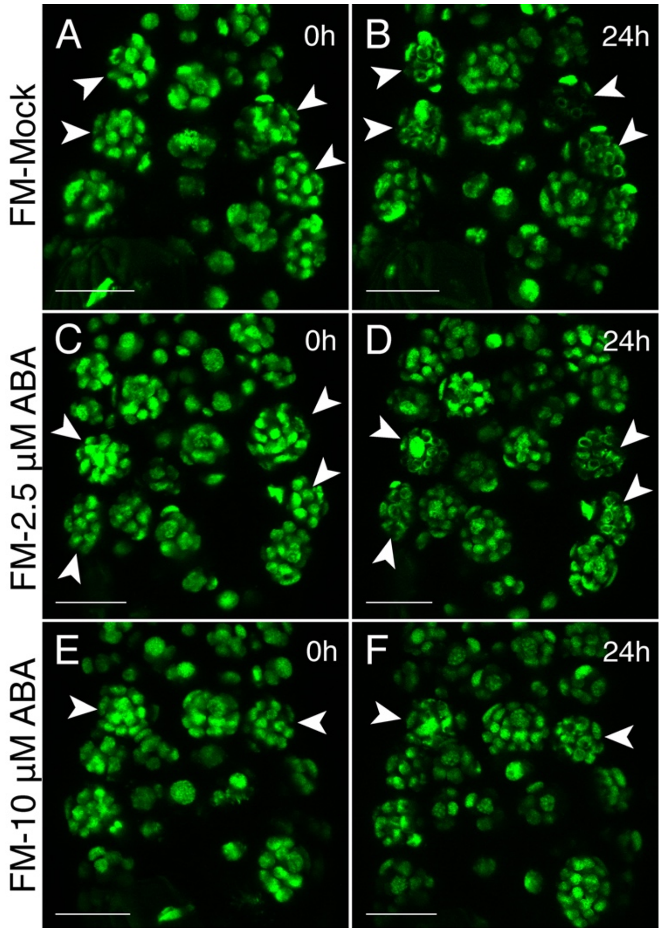

Supplementary Figure 13. Time-lapse confocal imaging reveals antheridium maturation in male gametophytes under different treatment conditions. (A-F) Transgenic Ceratopteris males expressing the nuclear marker *pCrUBQ10::H2B-GFP::3'CrUBQ10* were transferred to fresh FM supplemented with mock (A-B), 2.5 µM ABA (C-D), or 10 µM ABA (E-F), and imaged at 0 h and 24 h after transfer. White arrowheads indicate antheridia undergoing sperm differentiation, characterized by nuclei forming ring-like structures. Scale bar: 50 µm.

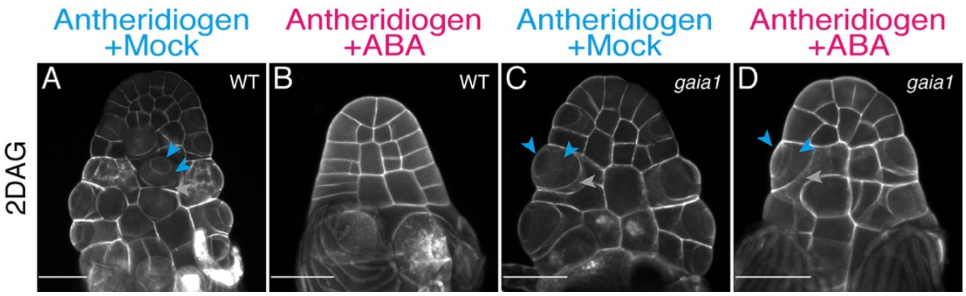

Supplementary Figure 14. Antheridium formation in the wild-type and *gaia1* mutant backgrounds. (A-D) Z-projection views of confocal images of male gametophytes (WT and *gaia1*) grown in the presence of antheridiogen alone or both antheridiogen and ABA. After germination on CFM, gametophytes (at 0 DAG) were transferred to CFM with mock treatment (A-B) or CFM supplemented with 2.5 μM ABA (C-D). Confocal images showing the cell outlines of gametophytes with the indicated genotypes and treatments at 2 DAG. One representative antheridium for each gametophyte (A, C, D) is highlighted, with their cap and ring cells labelled with blue arrowheads and the basal shield cell labeled with gray arrowheads. Scale bar: 50 μm.

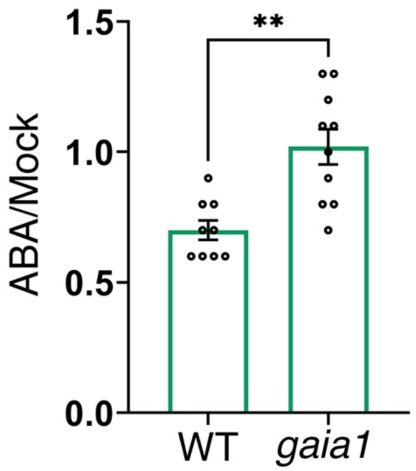

Supplementary Figure 15. Effects of antheridiogen and ABA on cell proliferation in WT and *gaia1* male gametophytes over an 18-hour period. Bar graph showing the relative effect of ABA vs. mock treatment on nucleus numbers in wild-type (WT) and *gaia1* male gametophytes following 18 h of treatment with antheridiogen plus ABA (n = 9 biologically independent samples for WT; n = 10 biologically independent samples for *gaia1*) or antheridiogen plus mock (n = 9 biologically independent samples for WT; n = 10 biologically independent samples for *gaia1*). Spores of WT and *gaia1* were plated on CFM plates. At 3 DAG, male gametophytes were randomly selected and transferred to CFM with mock or with 2.5 µM ABA. After 18 hours, samples were subjected to nuclear staining and imaged. Nucleus numbers were counted for non-antheridium cells and early-stage antheridia (each containing no more than seven nuclei) located in the apical half of the male gametophytes. For each genotype, nucleus number of each ABA-treated sample was normalized to the average nucleus number of the mock-treated samples. ** $p < 0.01$ (Two-tailed Student's t-test). Bars and error bars represent mean ± SEM (WT: 0.70 ± 0.04; *gaia1*: 1.02 ± 0.07). Source data are included in Supplementary Data 4.

Supplementary Movie 1. Complete Z-stack views of confocal images from one male gametophyte (Sample 9, shown in Fig. 4) from top to bottom at 0 h. Green indicates GFP-labelled nuclei captured from the GFP channel of the confocal image stacks. Purple dots represent the labels of each detected nucleus as presented in the computational lineage maps (shown in Fig. 7). Scale bar: 10 µm.

Supplementary Movie 2. Complete Z-stack views of confocal images from the male gametophyte (Sample 9, shown in Fig. 4) from top to bottom at 6 h. Green indicates GFP-labelled nuclei captured from the GFP channel of the confocal image stacks. Purple dots represent the labels of each detected nucleus as presented in the computational lineage maps (shown in Fig. 7). Scale bar: 10 μm.

Supplementary Movie 3. Complete Z-stack views of confocal images from the male gametophyte (Sample 9, shown in Fig. 4) from top to bottom at 12 h. Green indicates GFP-labelled nuclei captured from the GFP channel of the confocal image stacks. Purple dots represent the labels of each detected nucleus as presented in the computational lineage maps (shown in Fig. 7). Scale bar: 10 μm.

Supplementary Movie 4. Complete Z-stack views of confocal images from the male gametophyte (Sample 9, shown in Fig. 4) from top to bottom at 18 h. Green indicates GFP-labelled nuclei captured from the GFP channel of the confocal image stacks. Purple dots represent the labels of each detected nucleus as presented in the computational lineage maps (shown in Fig. 7). Scale bar: 10 μm.

Supplementary Movie 5. Complete Z-stack views of confocal images from one male gametophyte (Sample 9, shown in Fig. 4) from top to bottom at 24 h. Green indicates GFP-labelled nuclei captured from the GFP channel of the confocal image stacks. Purple dots represent the labels of each detected nucleus as presented in the computational lineage maps (shown in Fig. 7). Scale bar: 10 μm.

Supplementary Movie 6. Complete Z-stack views of confocal images from one male gametophyte (Sample 9, shown in Fig. 4) from top to bottom at 30 h. Green indicates GFP-labelled nuclei captured from the GFP channel of the confocal image stacks. Purple dots represent the labels of each detected nucleus as presented in the computational lineage maps (shown in Fig. 7). Scale bar: 10 μm.

Supplementary Movie 7. Complete Z-stack views of confocal images from one male gametophyte (Sample 9, shown in Fig. 4) from top to bottom at 36 h. Green indicates GFP-labelled nuclei captured from the GFP channel of the confocal image stacks. Purple dots represent the labels of each detected nucleus as presented in the computational lineage maps (shown in Fig. 7). Scale bar: 10 µm.

Supplementary Movie 8. 3D rotational view of the cell lineage map from the male gametophyte (sample 9 in Fig. 7) at 0 h. The Z projection view of the lineage map is shown in Fig. 7A. Each color-coded dot represents a nucleus in Fig. 4A. Scale bar: 20 µm.

Supplementary Movie 9. 3D rotational view of the cell lineage map from the male gametophyte (sample 9 in Fig. 7) at 6 h. The Z projection view of the cell lineage map is shown in Fig. 7B. Scale bar: 20 µm.

Supplementary Movie 10. 3D rotational view of the cell lineage map from the male gametophyte (sample 9 in Fig. 7) at 12 h. The Z projection view of the cell lineage map is shown in Fig. 7C, D. Scale bar: 20 µm.

Supplementary Movie 11. 3D rotational view of the cell lineage map from the male gametophyte (sample 9 in Fig. 7) at 18 h. The Z projection view of the cell lineage map is shown in Fig. 7E, F. Scale bar: 20 µm.

Supplementary Movie 12. 3D rotational view of the cell lineage map from the male gametophyte (sample 9 in Fig. 7) at 24 h. The Z projection view of the cell lineage map is shown in Fig. 7G, H. Scale bar: 20 µm.

Supplementary Movie 13. 3D rotational view of the cell lineage maps from the male gametophyte (sample 9 in Fig. 7) at 30 h. The Z projection view of the cell lineage map is shown in Fig. 7I, J. Scale bar: 20 µm.

Supplementary Movie 14. 3D rotational view of the cell lineage maps from the male gametophyte (sample 9 in Fig. 7) at 36 h. The Z projection view of the cell lineage map is shown in Fig. 7K, L. Scale bar: 20 μm.

Supplementary Movie 15. 3D rotational view of the cell division map of the male gametophyte (sample 9 in Fig. 4) from 0-12 hours. Green dots represent cells remained undivided, while magenta dots indicate cells underwent division during the 12-hour time period. The Z projection view of the cell division map is shown in Fig. 8A. Scale bar: 20 μm.

Supplementary Movie 16. 3D rotational view of the cell division map of the male gametophyte (sample 9 in Fig. 4) from 12-24 hours. Green dots represent cells remained undivided, while magenta dots indicate cells underwent division during the 12-hour time period. The Z projection view of the cell division map is shown in Fig. 8B. Scale bar: 20 μm.

Supplementary Movie 17. 3D rotational view of the cell division map of the male gametophyte (sample 9 in Fig. 4) from 24-36 hours. Green dots represent cells remained undivided, while magenta dots indicate cells underwent division during the 12-hour time period. The Z projection view of the cell division map is shown in Fig. 8C. Scale bar: 20 μm.

Supplementary Data 1. Source data for Fig. 9V.

Supplementary Data 2. Source data for Fig. 9W.

Supplementary Data 3. Source date for Supplementary Figure 12.

Supplementary Data 4. Source data for Supplementary Figure 15.

Supplementary Data 5. Code used for image analysis in this study.
