# Supplementary material for: Cell division and lineage dynamics during antheridium differentiation and male gametophyte development in Ceratopteris richardii
Source: Commun Biol. 2026 Apr 30;9:911. doi: 10.1038/s42003-026-10135-w (PMC13338035; doi:10.1038/s42003-026-10135-w)
Supplement: Supplementary file 2 — Description of Additional Supplementary Files [file 42003_2026_10135_MOESM2_ESM.docx]

**Description of Additional Supplementary File**

File name: Supplementary Data 1
Description: Source data for Fig. 9V.

File name: Supplementary Data 2
Description: Source data for Fig. 9W.

File name: Supplementary Data 3
Description: Source date for Supplementary Figure 12.

File name: Supplementary Data 4
Description: Source data for Supplementary Figure 15.

File name: Supplementary Data 5
Description: Code used for image analysis in this study.

File name: Supplementary Movie 1
Description: Complete Z-stack views of confocal images from one male gametophyte (Sample 9, shown in Fig. 4) from top to bottom at 0 h. Green indicates GFP-labelled nuclei captured from the GFP channel of the confocal image stacks. Purple dots represent the labels of each detected nucleus as presented in the computational lineage maps (shown in Fig. 7). Scale bar: 10 μm.

File name: Supplementary Movie 2
Description: Complete Z-stack views of confocal images from the male gametophyte (Sample 9, shown in Fig. 4) from top to bottom at 6 h. Green indicates GFP-labelled nuclei captured from the GFP channel of the confocal image stacks. Purple dots represent the labels of each detected nucleus as presented in the computational lineage maps (shown in Fig. 7). Scale bar: 10 μm.

File name: Supplementary Movie 3
Description: Complete Z-stack views of confocal images from the male gametophyte (Sample 9, shown in Fig. 4) from top to bottom at 12 h. Green indicates GFP-labelled nuclei captured from the GFP channel of the confocal image stacks. Purple dots represent the labels of each detected nucleus as presented in the computational lineage maps (shown in Fig. 7). Scale bar: 10 μm.

File name: Supplementary Movie 4
Description: Complete Z-stack views of confocal images from the male gametophyte (Sample 9, shown in Fig. 4) from top to bottom at 18 h. Green indicates GFP-labelled nuclei captured from the GFP channel of the confocal image stacks. Purple dots represent the labels of each detected nucleus as presented in the computational lineage maps (shown in Fig. 7). Scale bar: 10 μm.

File name: Supplementary Movie 5
Description: Complete Z-stack views of confocal images from one male gametophyte (Sample 9, shown in Fig. 4) from top to bottom at 24 h. Green indicates GFP-labelled nuclei captured from the GFP channel of the confocal image stacks. Purple dots represent the labels of each detected nucleus as presented in the computational lineage maps (shown in Fig. 7). Scale bar: 10 μm.

File name: Supplementary Movie 6
Description: Complete Z-stack views of confocal images from one male gametophyte (Sample 9, shown in Fig. 4) from top to bottom at 30 h. Green indicates GFP-labelled nuclei captured from the GFP channel of the confocal image stacks. Purple dots represent the labels of each detected nucleus as presented in the computational lineage maps (shown in Fig. 7). Scale bar: 10 μm.

File name: Supplementary Movie 7
Description: Complete Z-stack views of confocal images from one male gametophyte (Sample 9, shown in Fig. 4) from top to bottom at 36 h. Green indicates GFP-labelled nuclei captured from the GFP channel of the confocal image stacks. Purple dots represent the labels of each detected nucleus as presented in the computational lineage maps (shown in Fig. 7). Scale bar: 10 μm.

File name: Supplementary Movie 8
Description: 3D rotational view of the cell lineage map from the male gametophyte (sample 9 in Fig. 7) at 0 h. The Z projection view of the lineage map is shown in Fig. 7A. Each color-coded dot represents a nucleus in Fig. 4A. Scale bar: 20 μm.

File name: Supplementary Movie 9
Description: 3D rotational view of the cell lineage map from the male gametophyte (sample 9 in Fig. 7) at 6 h. The Z projection view of the cell lineage map is shown in Fig. 7B. Scale bar: 20 μm.

File name: Supplementary Movie 10
Description: 3D rotational view of the cell lineage map from the male gametophyte (sample 9 in Fig. 7) at 12 h. The Z projection view of the cell lineage map is shown in Fig. 7C, D. Scale bar: 20 μm.

File name: Supplementary Movie 11
Description: 3D rotational view of the cell lineage map from the male gametophyte (sample 9 in Fig. 7) at 18 h. The Z projection view of the cell lineage map is shown in Fig. 7E, F. Scale bar: 20 μm.

File name: Supplementary Movie 12
Description: 3D rotational view of the cell lineage map from the male gametophyte (sample 9 in Fig. 7) at 24 h. The Z projection view of the cell lineage map is shown in Fig. 7G, H. Scale bar: 20 μm.

File name: Supplementary Movie 13
Description: 3D rotational view of the cell lineage maps from the male gametophyte (sample 9 in Fig. 7) at 30 h. The Z projection view of the cell lineage map is shown in Fig. 7I, J. Scale bar: 20 μm.

File name: Supplementary Movie 14
Description: 3D rotational view of the cell lineage maps from the male gametophyte (sample 9 in Fig. 7) at 36 h. The Z projection view of the cell lineage map is shown in Fig. 7K, L. Scale bar: 20 μm.

File name: Supplementary Movie 15
Description: 3D rotational view of the cell division map of the male gametophyte (sample 9 in Fig. 4) from 0-12 hours. Green dots represent cells remained undivided, while magenta dots indicate cells underwent division during the 12-hour time period. The Z projection view of the cell division map is shown in Fig. 8A. Scale bar: 20 μm.

File name: Supplementary Movie 16
Description: 3D rotational view of the cell division map of the male gametophyte (sample 9 in Fig. 4) from 12-24 hours. Green dots represent cells remained undivided, while magenta dots indicate cells underwent division during the 12-hour time period. The Z projection view of the cell division map is shown in Fig. 8B. Scale bar: 20 μm.

File name: Supplementary Movie 17
Description: 3D rotational view of the cell division map of the male gametophyte (sample 9 in Fig. 4) from 24-36 hours. Green dots represent cells remained undivided, while magenta dots indicate cells underwent division during the 12-hour time period. The Z projection view of the cell division map is shown in Fig. 8C. Scale bar: 20 μm.
