# Supplementary figures and images for: Cell division and lineage dynamics during antheridium differentiation and male gametophyte development in Ceratopteris richardii
Source: Commun Biol. 2026 Apr 30;9:911. doi: 10.1038/s42003-026-10135-w (PMC13338035; doi:10.1038/s42003-026-10135-w)

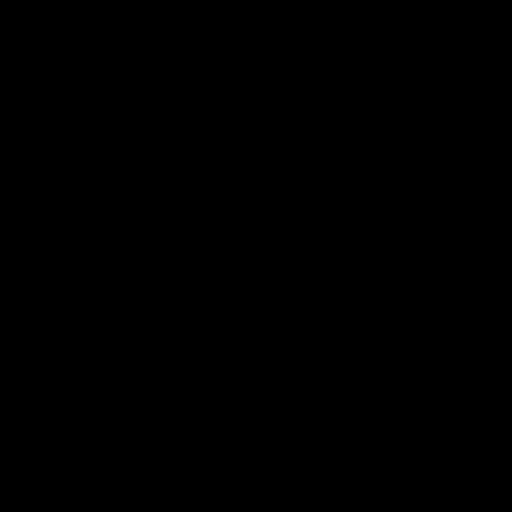

Supplement: Supplementary file 7 — Supplementary Data 5 [file 42003_2026_10135_MOESM7_ESM.zip › Supplementary Resource Code Package/3_Two Colors Cell Divisions/Sample2 0h 12h Two Colors Cell Divisions/CellDivisionsTwoColors_sample2 on CFM original 12h 255_Ball_Label.tif]

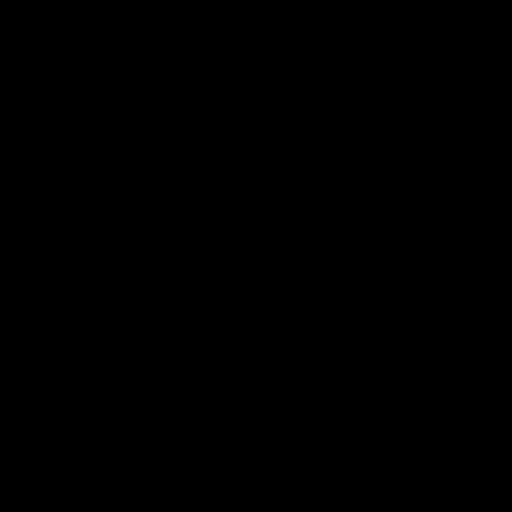

Supplement: Supplementary file 7 — Supplementary Data 5 [file 42003_2026_10135_MOESM7_ESM.zip › Supplementary Resource Code Package/3_Two Colors Cell Divisions/Sample2 0h 12h Two Colors Cell Divisions/sample2 on CFM original 0h 255_Ball_Label_New.tif]

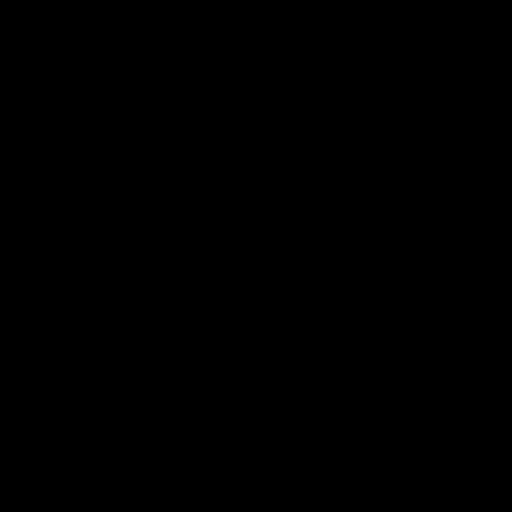

Supplement: Supplementary file 7 — Supplementary Data 5 [file 42003_2026_10135_MOESM7_ESM.zip › Supplementary Resource Code Package/3_Two Colors Cell Divisions/Sample2 0h 12h Two Colors Cell Divisions/sample2 on CFM original 12h 255_Ball_Label.tif]

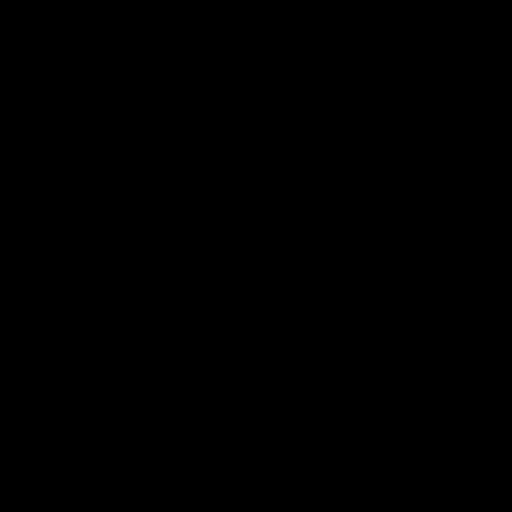

Supplement: Supplementary file 7 — Supplementary Data 5 [file 42003_2026_10135_MOESM7_ESM.zip › Supplementary Resource Code Package/3_Two Colors Cell Divisions/Sample2 0h 12h Two Colors Cell Divisions/CellDivisionsTwoColors_sample2 on CFM original 0h 255_Ball_Label.tif]

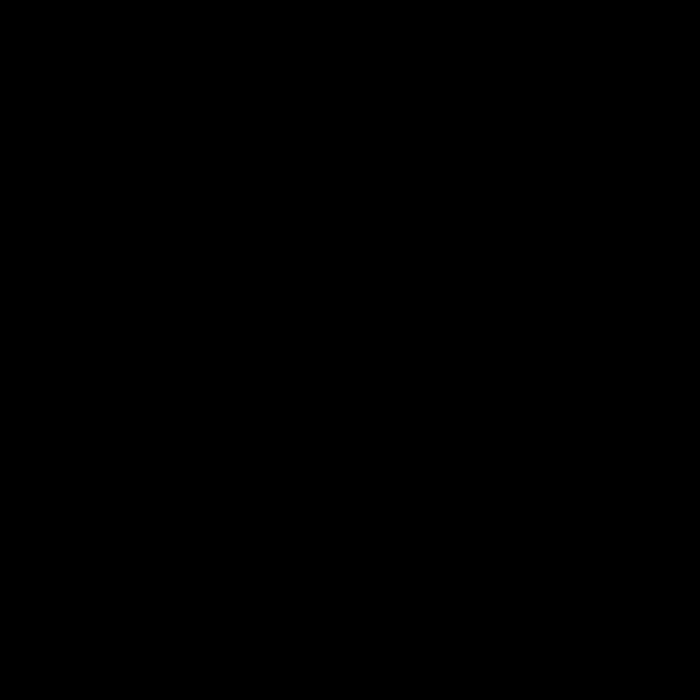

Supplement: Supplementary file 7 — Supplementary Data 5 [file 42003_2026_10135_MOESM7_ESM.zip › Supplementary Resource Code Package/1_Nuclei Detection and Corrections/Sample2_30h/sample2 on CFM original 30h 255_Label.tif]

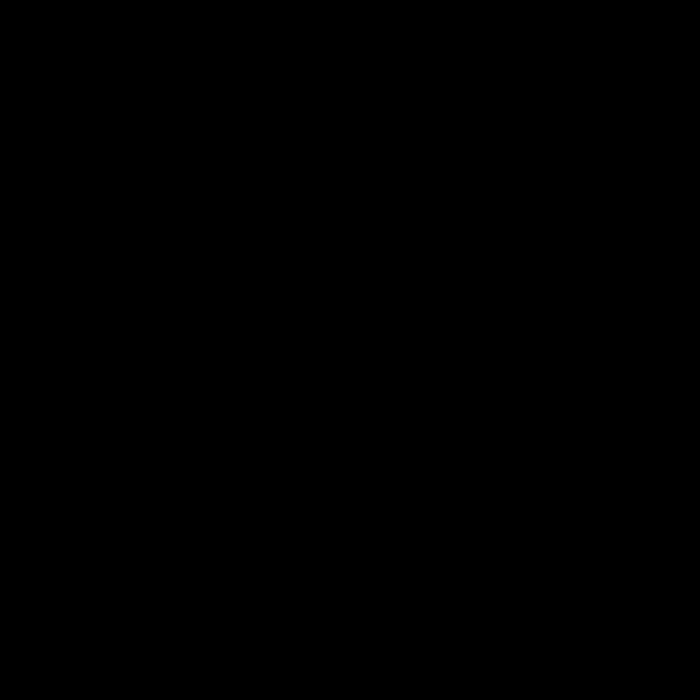

Supplement: Supplementary file 7 — Supplementary Data 5 [file 42003_2026_10135_MOESM7_ESM.zip › Supplementary Resource Code Package/1_Nuclei Detection and Corrections/Sample2_30h/sample2 on CFM original 30h_New_Ball_Colorful.tif]

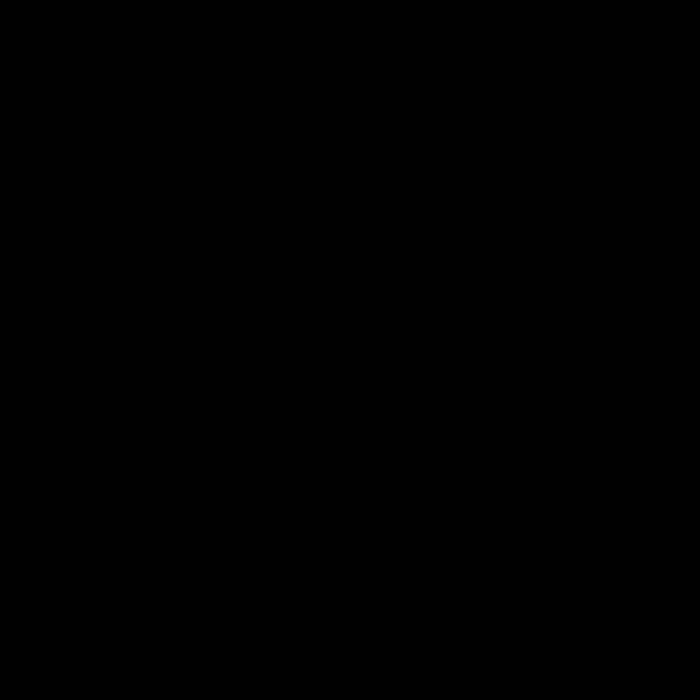

Supplement: Supplementary file 7 — Supplementary Data 5 [file 42003_2026_10135_MOESM7_ESM.zip › Supplementary Resource Code Package/1_Nuclei Detection and Corrections/Sample2_30h/sample2 on CFM original 30h 255_Ball_Colorful.tif]

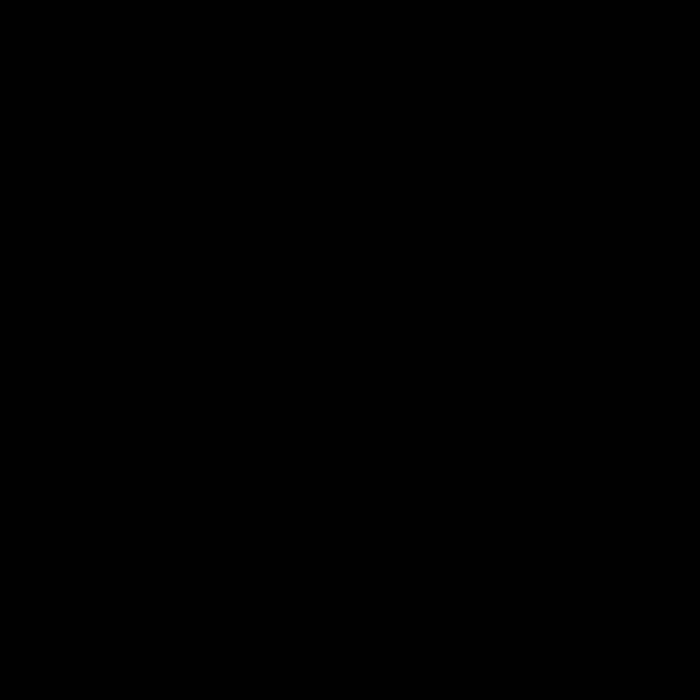

Supplement: Supplementary file 7 — Supplementary Data 5 [file 42003_2026_10135_MOESM7_ESM.zip › Supplementary Resource Code Package/1_Nuclei Detection and Corrections/Sample2_30h/sample2 on CFM original 30h_New_Ball_Label.tif]

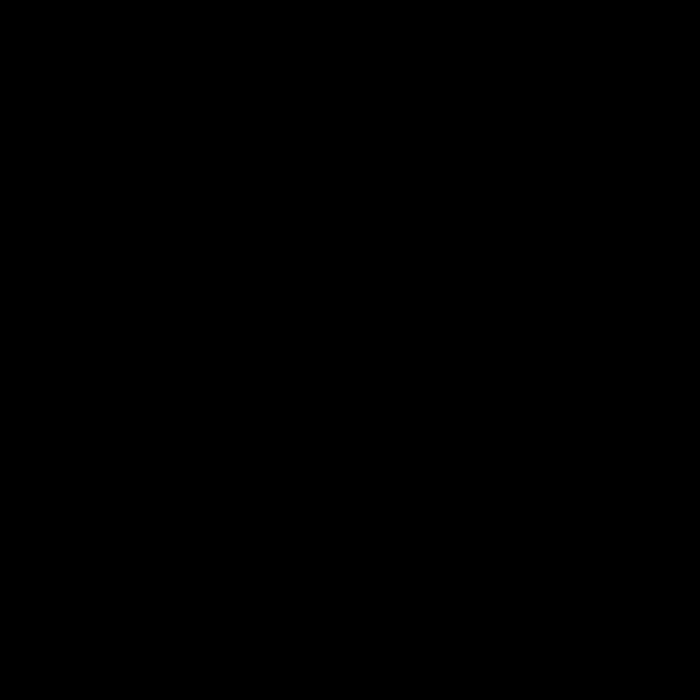

Supplement: Supplementary file 7 — Supplementary Data 5 [file 42003_2026_10135_MOESM7_ESM.zip › Supplementary Resource Code Package/1_Nuclei Detection and Corrections/Sample2_30h/sample2 on CFM original 30h 255_Ball_Label_DeletionNuclei.tif]

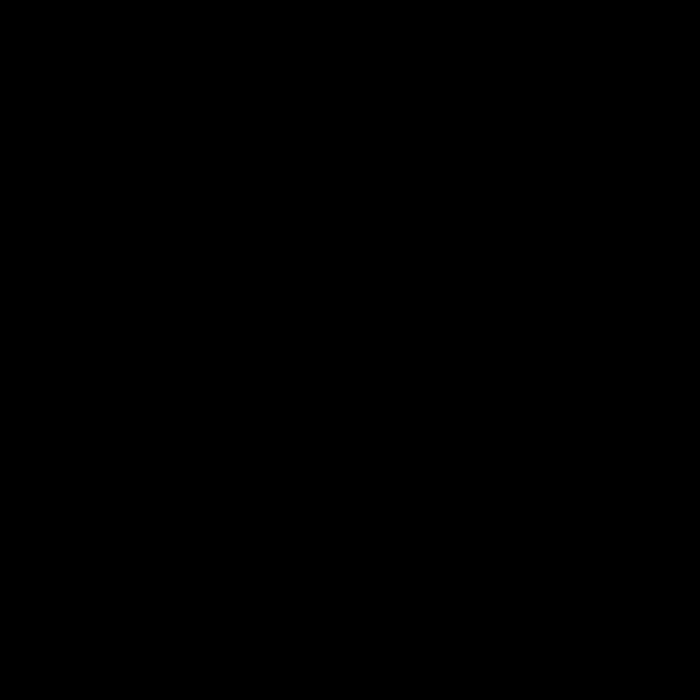

Supplement: Supplementary file 7 — Supplementary Data 5 [file 42003_2026_10135_MOESM7_ESM.zip › Supplementary Resource Code Package/1_Nuclei Detection and Corrections/Sample2_30h/sample2 on CFM original 30h 255_Ball_Label.tif]

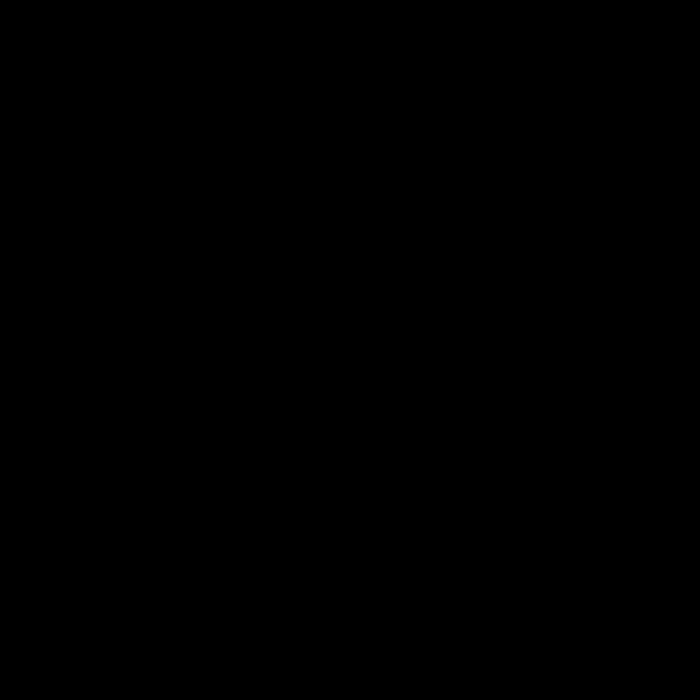

Supplement: Supplementary file 7 — Supplementary Data 5 [file 42003_2026_10135_MOESM7_ESM.zip › Supplementary Resource Code Package/1_Nuclei Detection and Corrections/Sample2_30h/sample2 on CFM original 30h 255_BW.tif]

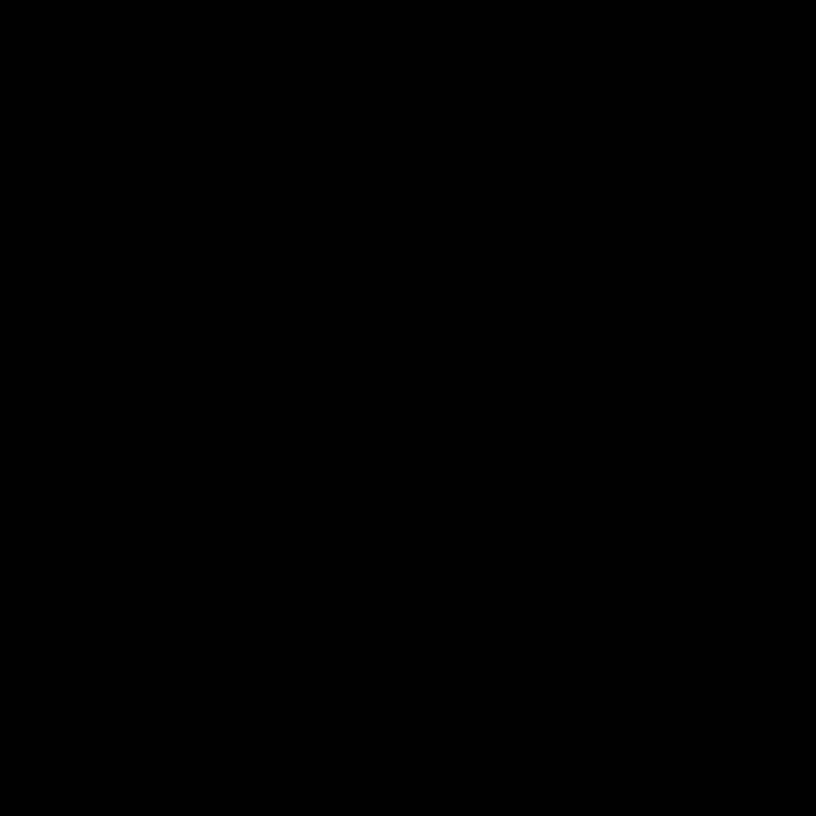

Supplement: Supplementary file 7 — Supplementary Data 5 [file 42003_2026_10135_MOESM7_ESM.zip › Supplementary Resource Code Package/4_Quantification of Cell Divisions/Sample7 Mock Colorful Cell Divisions Quantification/sample7 on CFM-mock 18h_New_Ball_Label.tif]

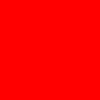

Supplement: Supplementary file 7 — Supplementary Data 5 [file 42003_2026_10135_MOESM7_ESM.zip › Supplementary Resource Code Package/4_Quantification of Cell Divisions/Sample7 Mock Colorful Cell Divisions Quantification/ColorBar max5/Bar_5.tif]

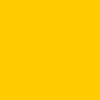

Supplement: Supplementary file 7 — Supplementary Data 5 [file 42003_2026_10135_MOESM7_ESM.zip › Supplementary Resource Code Package/4_Quantification of Cell Divisions/Sample7 Mock Colorful Cell Divisions Quantification/ColorBar max5/Bar_4.tif]

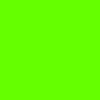

Supplement: Supplementary file 7 — Supplementary Data 5 [file 42003_2026_10135_MOESM7_ESM.zip › Supplementary Resource Code Package/4_Quantification of Cell Divisions/Sample7 Mock Colorful Cell Divisions Quantification/ColorBar max5/Bar_3.tif]

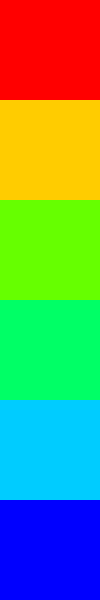

Supplement: Supplementary file 7 — Supplementary Data 5 [file 42003_2026_10135_MOESM7_ESM.zip › Supplementary Resource Code Package/4_Quantification of Cell Divisions/Sample7 Mock Colorful Cell Divisions Quantification/ColorBar max5/Bar_Max5.tif]

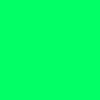

Supplement: Supplementary file 7 — Supplementary Data 5 [file 42003_2026_10135_MOESM7_ESM.zip › Supplementary Resource Code Package/4_Quantification of Cell Divisions/Sample7 Mock Colorful Cell Divisions Quantification/ColorBar max5/Bar_2.tif]

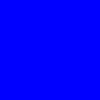

Supplement: Supplementary file 7 — Supplementary Data 5 [file 42003_2026_10135_MOESM7_ESM.zip › Supplementary Resource Code Package/4_Quantification of Cell Divisions/Sample7 Mock Colorful Cell Divisions Quantification/ColorBar max5/Bar_0.tif]

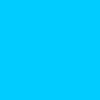

Supplement: Supplementary file 7 — Supplementary Data 5 [file 42003_2026_10135_MOESM7_ESM.zip › Supplementary Resource Code Package/4_Quantification of Cell Divisions/Sample7 Mock Colorful Cell Divisions Quantification/ColorBar max5/Bar_1.tif]

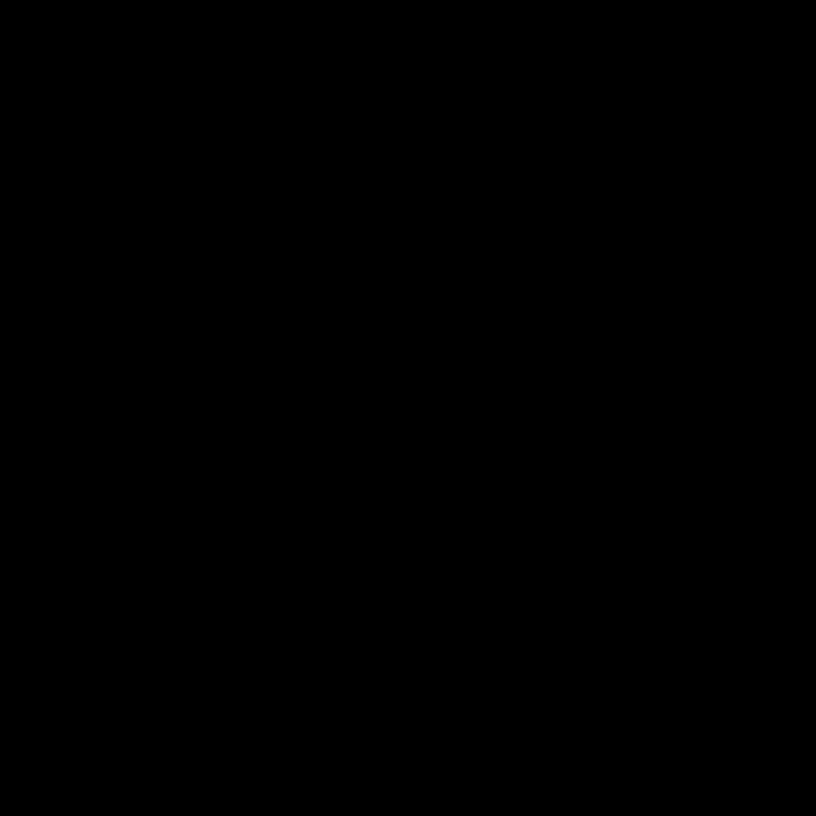

Supplement: Supplementary file 7 — Supplementary Data 5 [file 42003_2026_10135_MOESM7_ESM.zip › Supplementary Resource Code Package/4_Quantification of Cell Divisions/Sample7 Mock Colorful Cell Divisions Quantification/CellDivisionsQuantifications_sample7_Mock_0h_18h_0h_max5.tif]

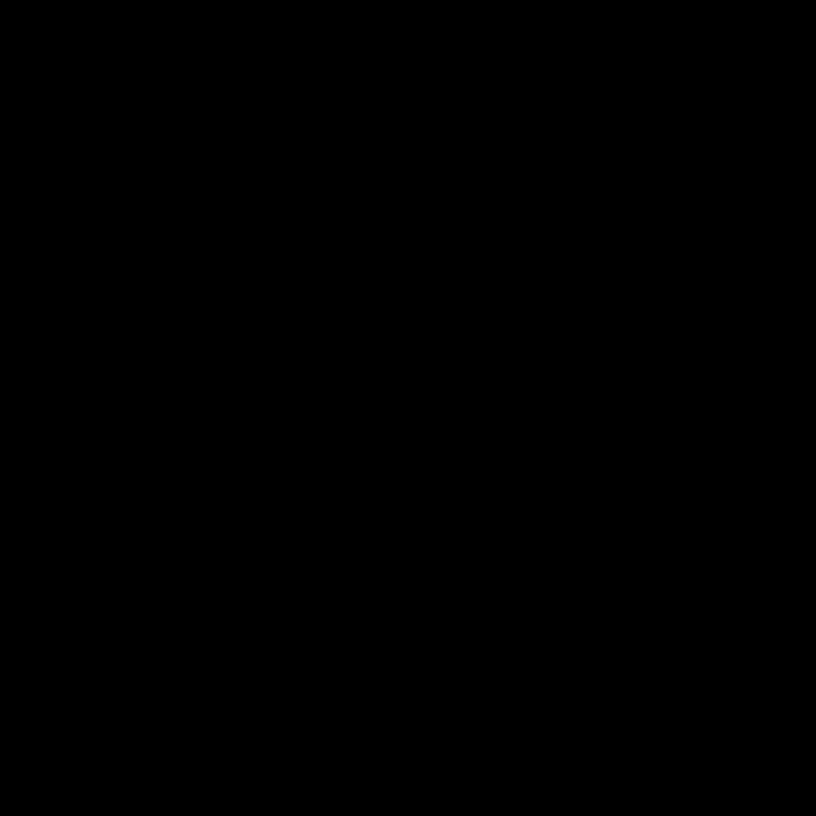

Supplement: Supplementary file 7 — Supplementary Data 5 [file 42003_2026_10135_MOESM7_ESM.zip › Supplementary Resource Code Package/4_Quantification of Cell Divisions/Sample7 Mock Colorful Cell Divisions Quantification/sample7 on CFM-mock 0h_New_Ball_Label.tif]

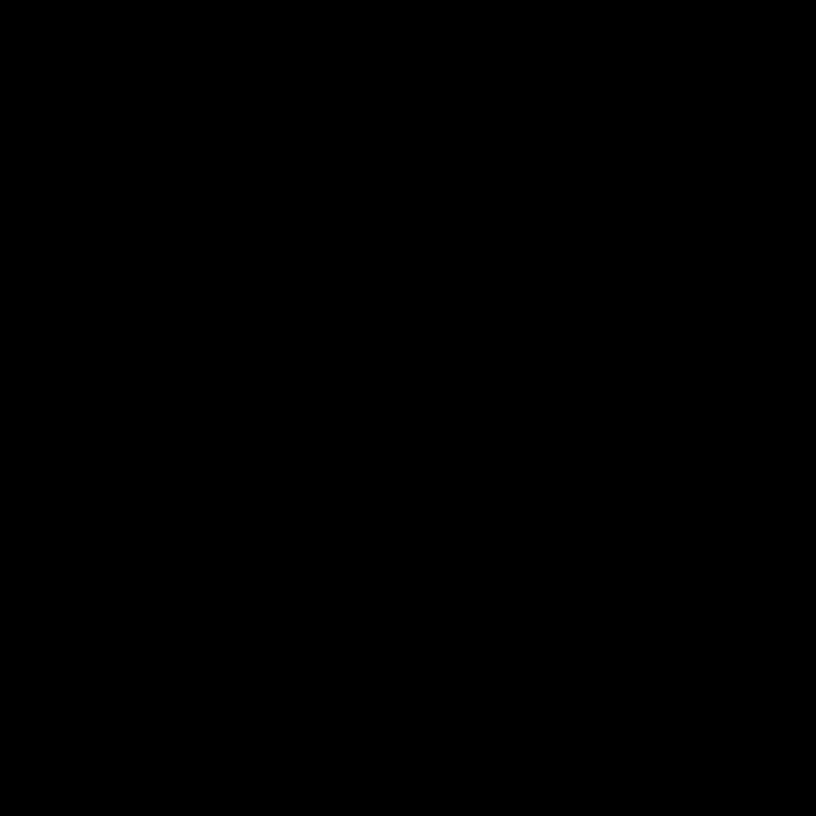

Supplement: Supplementary file 7 — Supplementary Data 5 [file 42003_2026_10135_MOESM7_ESM.zip › Supplementary Resource Code Package/4_Quantification of Cell Divisions/Sample7 Mock Colorful Cell Divisions Quantification/CellDivisionsQuantifications_sample7_Mock_0h_18h_18h_max5.tif]

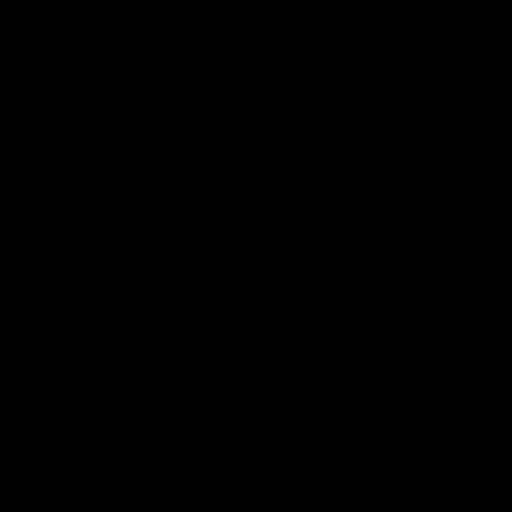

Supplement: Supplementary file 7 — Supplementary Data 5 [file 42003_2026_10135_MOESM7_ESM.zip › Supplementary Resource Code Package/2_Colorful Cell Lineage Generation/Sample2 Color Lineage/Sample2_6h_12h Lineage/sample2 on CFM original 6h 255_Ball_Label.tif]

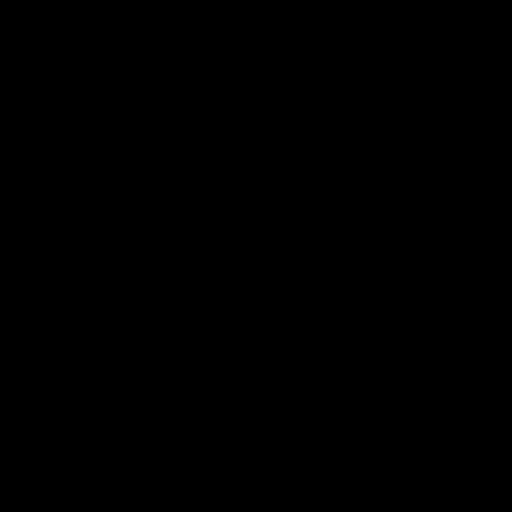

Supplement: Supplementary file 7 — Supplementary Data 5 [file 42003_2026_10135_MOESM7_ESM.zip › Supplementary Resource Code Package/2_Colorful Cell Lineage Generation/Sample2 Color Lineage/Sample2_6h_12h Lineage/Colorful_sample2 on CFM original 6h 255_Ball_Label.tif]

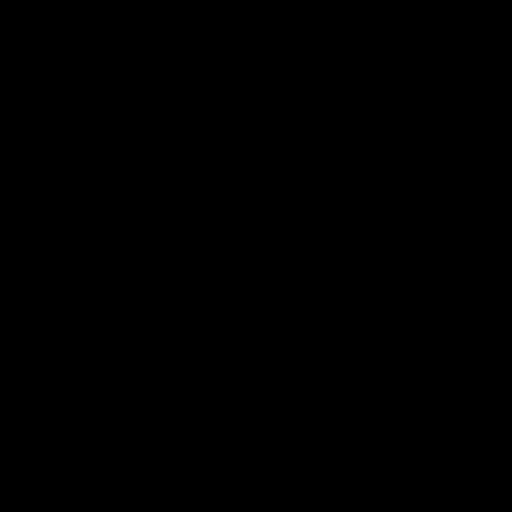

Supplement: Supplementary file 7 — Supplementary Data 5 [file 42003_2026_10135_MOESM7_ESM.zip › Supplementary Resource Code Package/2_Colorful Cell Lineage Generation/Sample2 Color Lineage/Sample2_6h_12h Lineage/Colorful_sample2 on CFM original 12h 255_Ball_Label.tif]

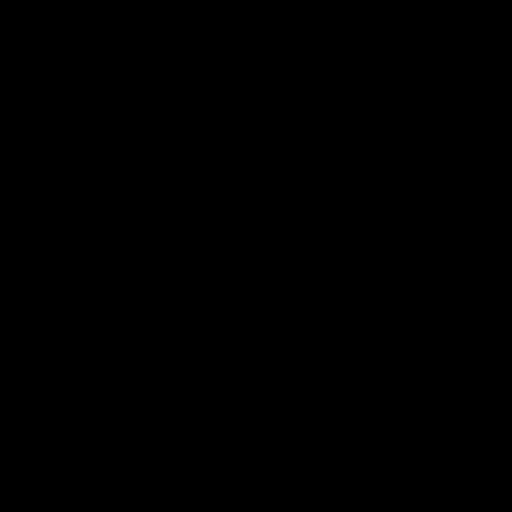

Supplement: Supplementary file 7 — Supplementary Data 5 [file 42003_2026_10135_MOESM7_ESM.zip › Supplementary Resource Code Package/2_Colorful Cell Lineage Generation/Sample2 Color Lineage/Sample2_30h_36h Lineage/Colorful_sample2 on CFM original 36h 255_Ball_Label.tif]

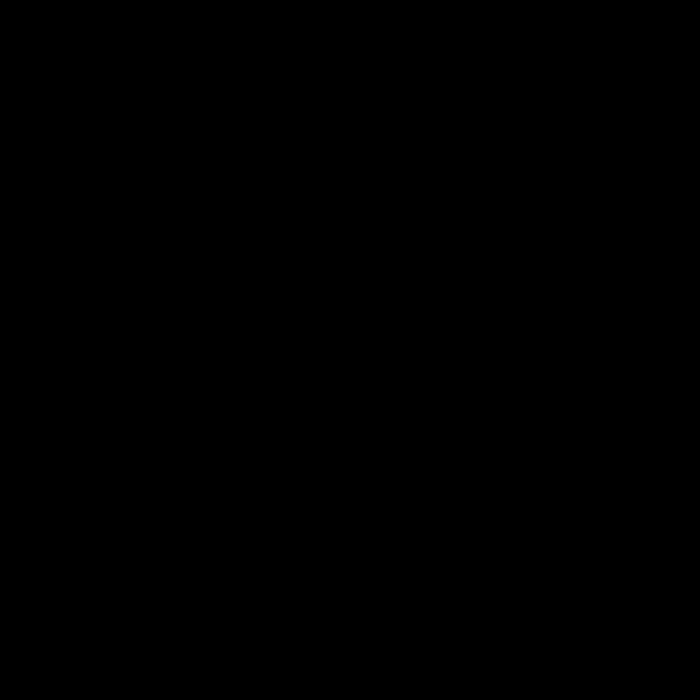

Supplement: Supplementary file 7 — Supplementary Data 5 [file 42003_2026_10135_MOESM7_ESM.zip › Supplementary Resource Code Package/2_Colorful Cell Lineage Generation/Sample2 Color Lineage/Sample2_30h_36h Lineage/Colorful_sample2 on CFM original 30h 255_Ball_Label.tif]

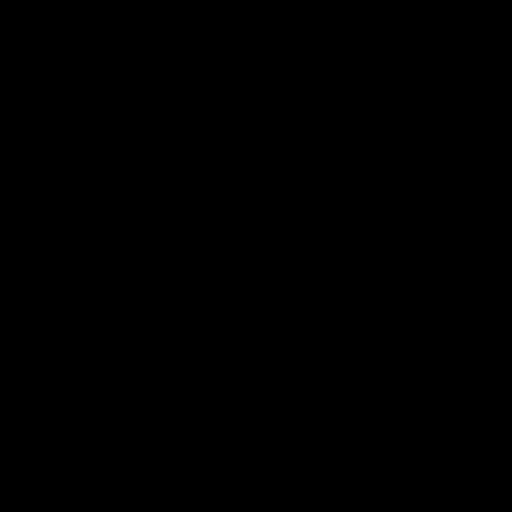

Supplement: Supplementary file 7 — Supplementary Data 5 [file 42003_2026_10135_MOESM7_ESM.zip › Supplementary Resource Code Package/2_Colorful Cell Lineage Generation/Sample2 Color Lineage/Sample2_30h_36h Lineage/sample2 on CFM original 36h_Ball_Label_New.tif]

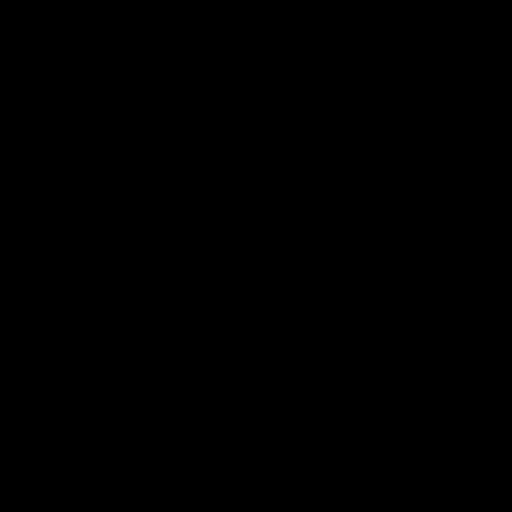

Supplement: Supplementary file 7 — Supplementary Data 5 [file 42003_2026_10135_MOESM7_ESM.zip › Supplementary Resource Code Package/2_Colorful Cell Lineage Generation/Sample2 Color Lineage/Sample2_12h_18h Lineage/Colorful_sample2 on CFM original 18h 255_Ball_Label.tif]

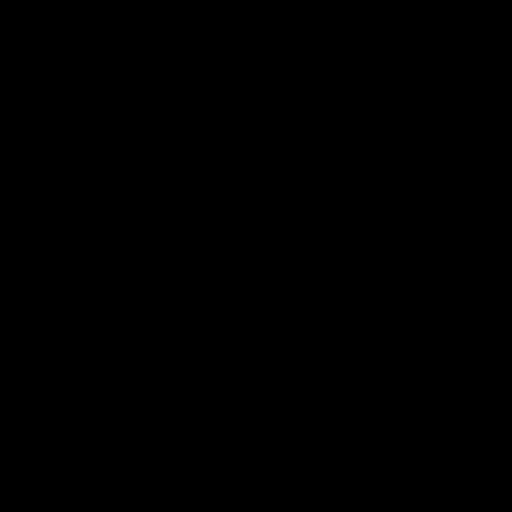

Supplement: Supplementary file 7 — Supplementary Data 5 [file 42003_2026_10135_MOESM7_ESM.zip › Supplementary Resource Code Package/2_Colorful Cell Lineage Generation/Sample2 Color Lineage/Sample2_12h_18h Lineage/sample2 on CFM original 18h 255_Ball_Label_New.tif]

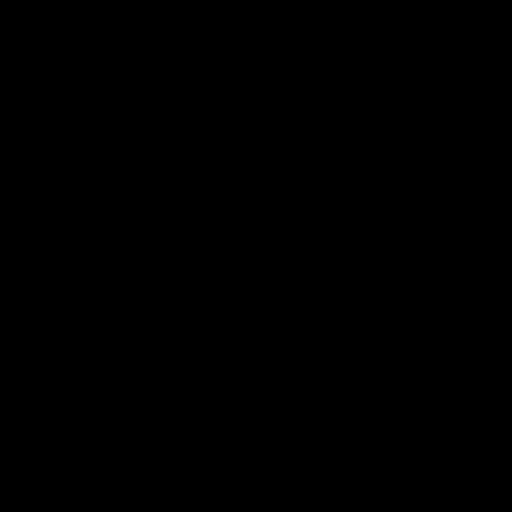

Supplement: Supplementary file 7 — Supplementary Data 5 [file 42003_2026_10135_MOESM7_ESM.zip › Supplementary Resource Code Package/2_Colorful Cell Lineage Generation/Sample2 Color Lineage/Sample2_24h_30h Lineage/Colorful_sample2 on CFM original 24h 255_Ball_Label.tif]

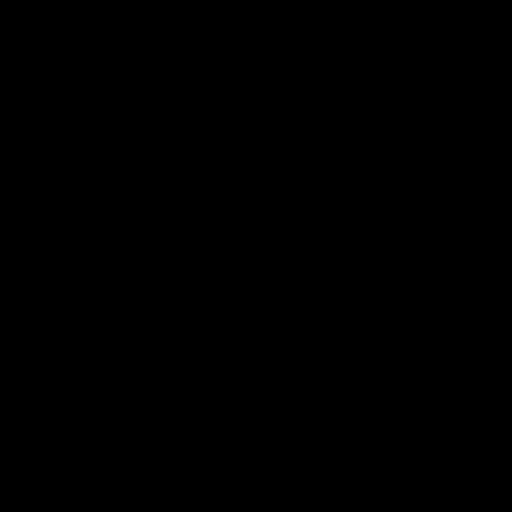

Supplement: Supplementary file 7 — Supplementary Data 5 [file 42003_2026_10135_MOESM7_ESM.zip › Supplementary Resource Code Package/2_Colorful Cell Lineage Generation/Sample2 Color Lineage/Sample2_24h_30h Lineage/sample2 on CFM original 24h_Ball_Label_New.tif]

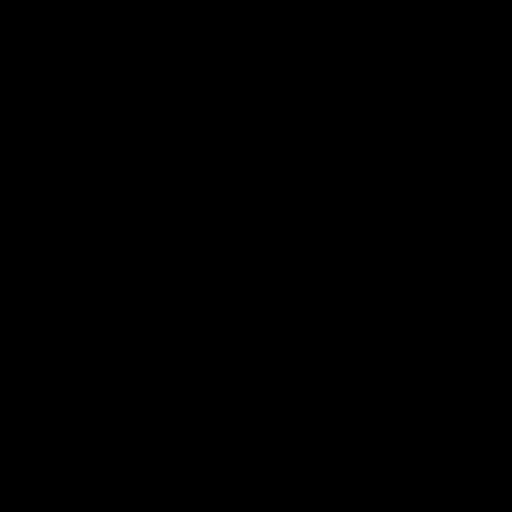

Supplement: Supplementary file 7 — Supplementary Data 5 [file 42003_2026_10135_MOESM7_ESM.zip › Supplementary Resource Code Package/2_Colorful Cell Lineage Generation/Sample2 Color Lineage/Sample2_Colorful Lineage Results/Colorful_sample2 on CFM original 0h 255_Ball_Label.tif]

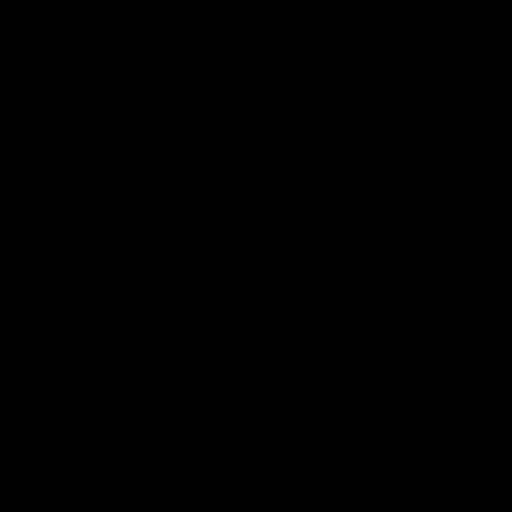

Supplement: Supplementary file 7 — Supplementary Data 5 [file 42003_2026_10135_MOESM7_ESM.zip › Supplementary Resource Code Package/2_Colorful Cell Lineage Generation/Sample2 Color Lineage/Sample2_0h_6h Lineage/sample2 on CFM original 0h 255_Ball_Label_Edited.tif]

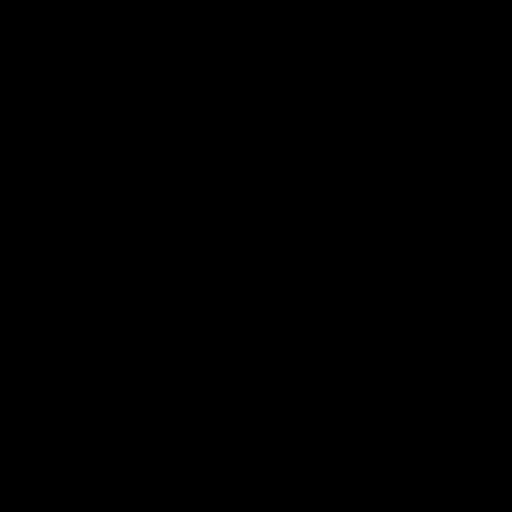

Supplement: Supplementary file 7 — Supplementary Data 5 [file 42003_2026_10135_MOESM7_ESM.zip › Supplementary Resource Code Package/2_Colorful Cell Lineage Generation/Sample2 Color Lineage/Sample2_0h_6h Lineage/sample2 on CFM original 6h 255_Ball_Label_Edited.tif]
